# The Biomodification and Biomimetic Synthesis of 2D Nanomaterial-Based Nanohybrids for Biosensor Applications: A Review

**DOI:** 10.3390/bios15050328

**Published:** 2025-05-20

**Authors:** Ranran Wang, Xinyue Wang, Yan Wang, Gang Wei

**Affiliations:** 1 College of Chemistry and Chemical Engineering, Qingdao University, 266071 Qingdao, China; wangranran0110@outlook.com (R.W.); xinyuewang0216@outlook.com (X.W.); 2 Key Laboratory of Rubber-Plastics, Ministry of Education/Shandong Provincial Key Laboratory of Rubber-Plastics, School of Polymer Science and Engineering, Qingdao University of Science and Technology, 266042 Qingdao, China

**Keywords:** 2DNMs, biomodification, biomimetic synthesis, nanohybrids, biosensors

## Abstract

Two-dimensional nanomaterials (2DNMs) exhibit significant potential for the development of functional and specifically targeted biosensors, owing to their unique planar nanosheet structures and distinct physical and chemical properties. Biomodification and biomimetic synthesis offer green and mild approaches for the fabrication of multifunctional nanohybrids with enhanced catalytic, fluorescent, electronic, and optical properties, thereby expanding their utility in constructing high-performance biosensors. In this review, we present recent advances in the synthesis of 2DNM-based nanohybrids via both biomodification and biomimetic strategies for biosensor applications. We discuss covalent and non-covalent biomodification methods involving various biomolecules, including peptides, proteins, DNA/RNA, enzymes, biopolymers, and bioactive polysaccharides. The engineering of biomolecule–nanomaterial interfaces for the creation of biomodified 2DNM-based nanohybrids is also explored. Furthermore, we summarize the biomimetic synthesis of 2DNM-based bio–nanohybrids through pathways such as bio-templating, biomolecule-directed self-assembly, biomineralization, and biomimetic functional integration. The potential applications of these nanohybrids in diverse biosensing platforms—including colorimetric, surface plasmon resonance, electrochemical, fluorescence, photoelectrochemical, and integrated multimodal biosensors—are introduced and discussed. Finally, we analyze the opportunities and challenges associated with this rapidly developing field. We believe this comprehensive review will provide valuable insights into the biofunctionalization of 2DNMs and guide the rational design of advanced biosensors for diagnostic applications.

## 1. Introduction

Two-dimensional nanomaterials (2DNMs), defined by their atomic-scale thickness, exceptionally high specific surface areas, and quantum-confined domains, constitute a burgeoning class of advanced functional materials. Driven by rapid advances in nanotechnology and the life sciences, 2DNMs have garnered intense research interest owing to their unique structural features, superior physicochemical characteristics, and broad potential across multiple disciplines [1,2]. A pivotal development in this domain was the formulation of the graphene oxide (GO) research paradigm, which marked a transformative shift in 2DNM science [3,4]. The mono- or few-layer architectures of 2DNMs afford them vastly greater surface-to-volume ratios relative to their bulk counterparts, thereby enhancing molecular and ionic adsorption and furnishing abundant active sites for subsequent functional modifications [5,6]. For example, GO combines exceptional electrical conductivity, mechanical robustness, thermal transport properties, and ultra-high carrier mobility, rendering it an ideal substrate for the fabrication of high-performance sensors and microelectronic components. Likewise, other prototypical 2DNMs such as transition metal dichalcogenides (e.g., MoS_2_, WS_2_) and black phosphorus (BP) have demonstrated outstanding efficacy in photocatalytic reactions, photothermal therapy, and bio-integrated technologies [7,8,9,10,11]. Collectively, the remarkable attributes of 2DNMs portend revolutionary applications in electronics, catalysis, photovoltaic energy conversion, and biomedicine [12,13]. In particular, their pronounced surface reactivity and tunable interfacial chemistry make them especially promising for biosensing platforms, enabling highly sensitive and selective detection modalities that could transform early disease diagnostics, environmental monitoring, and the next generation of bioelectronic devices [14,15].

Nevertheless, the practical deployment of individual 2DNMs is often impeded by intrinsic limitations, including poor colloidal stability in physiological media, a propensity for aggregation, and potential cytotoxicity. To surmount these challenges, biomodification has emerged as a crucial strategy for augmenting 2DNM performance. By grafting or adsorbing natural biomacromolecules such as peptides, proteins, nucleic acids (DNA/RNA), enzymes, biopolymers, and bioactive polysaccharides onto 2DNM surfaces via covalent or non-covalent interactions, a multifunctional biocompatible coating is formed. This biofunctional 2DNM layer not only improved colloidal stability and biocompatibility but also imparted specific functionalities, including molecular recognition and signal amplification [16,17,18]. Empirical studies have demonstrated that biomodified 2DNMs exhibit superior bioactivity and expanded application potential, particularly in cellular imaging, targeted drug delivery, and electrochemical biosensing, relative to their unmodified analogues [19,20]. Concurrently, to meet escalating requirements for biocompatibility, stability, and molecular specificity, researchers have pursued the biomimetic engineering of 2DNMs. By emulating natural processes such as templated mineralization, molecular self-assembly, and biomineralization, this approach transcends the limitations of single-component systems, enabling multifunctional integration and enhanced material performance [21]. The synthesis of 2DNMs via biomimetic routes offers an environmentally benign, facile, and highly controllable pathway to fabricating nanomaterials with tailored structures and functions, thereby advancing the development of green and sustainable two-dimensional nanomaterials [16].

The exceptional physicochemical attributes of 2DNMs confer distinct advantages for biosensor engineering [22,23]. Their ultra-high specific surface areas, coupled with abundant surface functional moieties, facilitate dense biomolecule immobilization and thus enable ultrasensitive analyte recognition. Moreover, the superior photoconversion efficiencies and intrinsic high electrical conductivities of 2DNMs provide an inherent platform for signal transduction and amplification, yielding rapid and robust sensor responses [24,25]. To date, 2DNM-based biosensors have been realized across a spectrum of detection modalities including colorimetric, surface plasmon resonance (SPR), electrochemical, fluorescence, and photoelectrochemical techniques. In order to overcome the constraints associated with any single sensing mechanism and to enhance analytical accuracy, multimodal platforms that integrate colorimetric, electrochemical, and photoelectrical readouts have been developed. These hybrid systems enable simultaneous or sequential signal generation and cross-validation of trace analytes within complex matrices. By exploiting the complementary strengths of multiple detection schemes, 2DNM-enabled biosensors achieve heightened sensitivity and selectivity while paving the way for portable, real-time diagnostic devices tailored for environmental monitoring, clinical diagnostics, and food safety applications [26,27].

Over the past decade, concerted global research efforts have substantially advanced the synthesis, functionalization, and application of 2DNMs [28,29,30,31]. Both experimental investigations and theoretical modeling have confirmed that 2DNMs produced via chemical or physical routes possess outstanding mechanical robustness, exceptional electrical conductivity, and tunable optical characteristics. Moreover, biological modifications further enhance their biocompatibility and expand their functional repertoire. Numerous illustrative examples have been documented, encompassing single-step biomolecular grafting, biomimetic fabrication techniques, and the engineering of diverse sensor platforms including colorimetric, SPR, and electrochemical modalities [15,32]. For example, Han and co-workers systematically reviewed a variety of hybridization strategies for 2DNMs [33], while Yin et al. provided a comprehensive account of layered 2DNM architectures tailored for photoelectrochemical sensing [34]. Likewise, Prodromidis and colleagues surveyed the synthesis methodologies and sensor applications of 2DNMs in electrochemical detection schemes [35]. Despite these valuable contributions, the literature on the biofunctionalization, biomimetic synthesis, and biosensor integration of 2DNMs remains dispersed and lacks a unified framework for comparing the relative merits of different approaches. This fragmentation hinders the establishment of cohesive design principles and theoretical underpinnings necessary for guiding future innovation. To address this gap, the present review delivers a systematic and in-depth analysis of biomodification techniques, biomimetic synthesis strategies, and the spectrum of biosensor applications enabled by 2DNMs, with the goal of furnishing new insights and theoretical support for the continued evolution of this dynamic field.

**Scheme 1:**
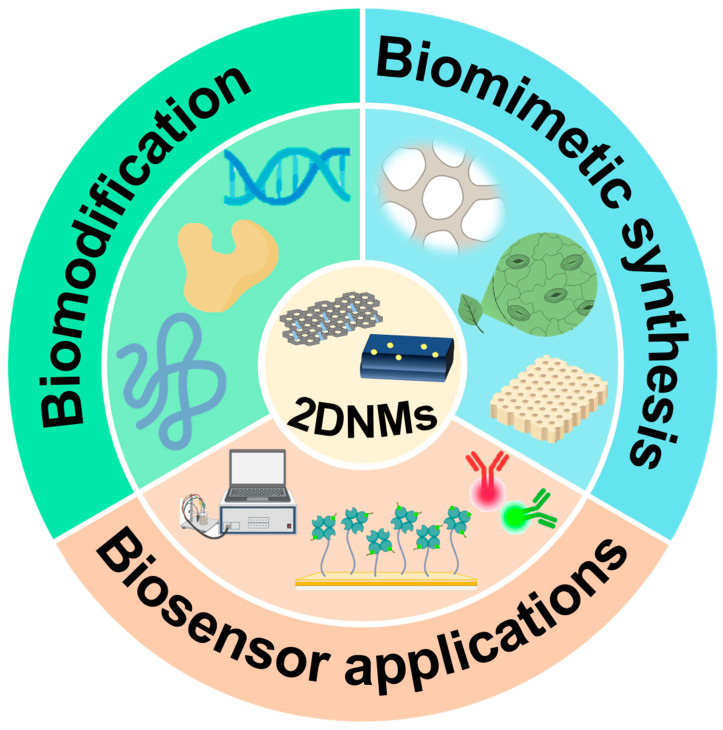
Schematic representation of biomodification and biomimetic synthesis of 2DNMs for biosensing.

In this review, we present a comprehensive and systematic overview of the advancements in 2DNMs, as shown in Scheme 1, encompassing their intrinsic physicochemical properties, functional modification strategies, biomimetic synthesis methodologies, and their integration into multimodal biosensing platforms. The objective is to provide theoretical insights and strategic guidance for the continued convergence of 2DNMs with biomedical technologies while also delineating prospective avenues for future technological innovation. Initially, we examine a broad range of surface modification strategies involving biomacromolecules such as peptides, proteins, nucleic acids (DNA/RNA), enzymes, biopolymers, and bioactive polysaccharides. These modifications are analyzed in the context of their mechanistic contributions to enhancing biocompatibility, physicochemical stability, and molecular-targeting capabilities. Subsequently, we explore biomimetic synthesis approaches including bio-templating, biologically directed molecular self-assembly, and biomineralization with a focus on recent progress in achieving precise structural control and multifunctional integration. Further, we investigate how the engineered surface functionalities and inherent signal amplification properties of 2DNMs facilitate the ultrasensitive detection of diverse biomarkers. This review covers a spectrum of sensor modalities, including colorimetric, SPR, electrochemical, fluorescence, photoelectrochemical, and integrated multimodal platforms. Finally, we present a forward-looking perspective on emerging trends in the field, such as environmentally sustainable synthesis approaches, stimuli-responsive smart materials, and the development of fully integrated multimodal detection systems. As nanotechnology and biotechnology increasingly intersect, 2DNMs are anticipated to play a pivotal role in advancing precision diagnostics, environmental monitoring, and intelligent sensing, thereby contributing significantly to human health and sustainable technological development.

## 2. Biomodification of 2DNMs

Individual 2DNMs often exhibit inherent physicochemical limitations that hinder their broader application potential. For example, BP possesses excellent photothermal conversion efficiency but is highly susceptible to oxidative degradation in aqueous environments. Similarly, graphitic carbon nitride (g-C_3_N_4_) exhibits promising photocatalytic activity; however, it suffers from a high rate of photogenerated charge carrier recombination and demonstrates potential cytotoxicity in biological contexts. To mitigate these limitations, rational surface modification strategies have been employed to enhance the stability, safety, and functionality of 2DNMs. For instance, polyethylene glycol (PEG) functionalization, known as PEGylation, and biomimetic membrane coatings have been shown to significantly improve the biocompatibility and reduce the cytotoxicity of g-C_3_N_4_. A wide array of biomaterials is utilized for the modification of 2DNMs, including peptides, proteins, nucleic acids (DNA/RNA), bio-enzymes, biodegradable polymers, and bioactive polysaccharides. These biointerfaces not only address material-specific deficiencies but also impart additional functionalities such as improved biostability, target specificity, and enhanced bioactivity, thereby expanding the practical utility of 2DNMs in biomedical and environmental applications.

The surface functionalization of 2DNMs via covalent or non-covalent integrations with other molecular entities enables the modulation of their surface characteristics, thereby conferring both the intrinsic properties of the nanomaterial and the specific functionalities of the modifying agents. Two primary strategies are currently employed for this purpose: covalent functionalization and non-covalent interaction. Covalent functionalization involves the formation of stable chemical bonds between the 2DNM surface and functional groups. This is typically achieved through reactions such as amide bond formation via carboxyl–amine condensation, thiol–maleimide coupling, and various click chemistry approaches. In contrast, non-covalent modification leverages weaker, reversible interactions including π–π stacking, electrostatic adsorption, van der Waals forces, and hydrogen bonding which preserve the structural integrity of the 2DNM while imparting functional versatility [36,37,38]. When biomolecules are employed as functionalization agents through either covalent or non-covalent routes, they significantly influence the physicochemical properties of 2DNMs. These biofunctionalized hybrids inherit the properties of both the 2D scaffold and the conjugated biomolecules, leading to enhanced biocompatibility, improved bioactivity, reduced cytotoxicity, and selective recognition capabilities toward specific cells or tissues. Such modifications effectively address the inherent limitations of unmodified 2DNMs and broaden their applicability across biomedical and environmental domains [39]. Therefore, the identification and development of suitable biomodifiers remain critical challenges in optimizing the performance and expanding the functional utility of 2DNMs in advanced applications.

### 2.1. Peptides

Peptides, which are polymers of amino acids connected by amide (peptide) bonds, serve as fundamental components in the physiological processes of living organisms. Long-chain peptides typically present an abundance of intermolecular interaction sites, including hydrogen bonds and hydrophobic domains, which contribute to enhanced interfacial adhesion with 2DNMs. Specifically, α-helical peptides, due to their stable helical structure and rigid backbone, can self-assemble into well-ordered nanofiber arrays on the surfaces of 2D materials, thereby improving the mechanical robustness of the resulting composites. In contrast, β-sheet peptides feature extended, planar architectures with high specific surface areas, which facilitate strong π-π stacking interactions with GO and other aromatic substrates, leading to significantly enhanced interfacial binding affinity [40,41]. They possess several advantageous characteristics, including high structural versatility, excellent biocompatibility, favorable biodegradability, and tunable morphological features. In the context of 2D peptide-based nanomaterials, their inherently large specific surface area and abundance of surface functional groups facilitate efficient interactions, both covalent and non-covalent, with a wide range of other materials. These properties make 2D peptide nanostructures highly promising candidates for the design and fabrication of advanced biomaterials and medical applications [42].

**Figure 1:**
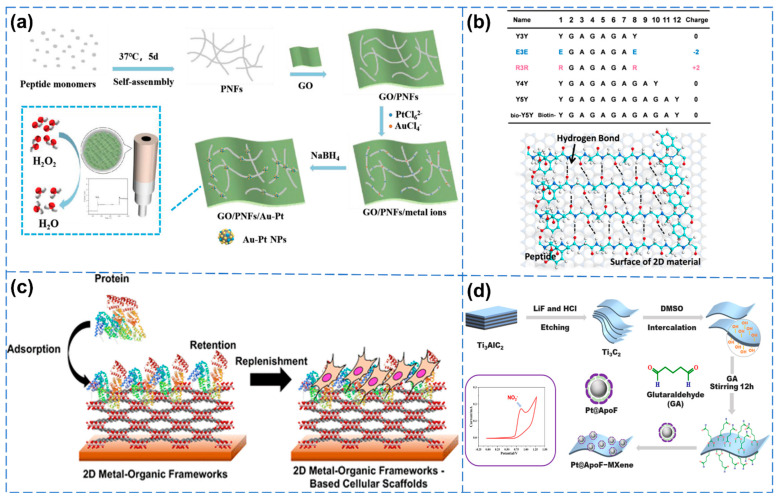
Modification of non-biological materials by peptide materials: (**a**) GO is modified by peptide nanofibers through non-covalent interaction. Reprinted with permission from ref. [45], Copyright 2021, Wiley-VCH. (**b**) Peptides self-assemble on the surface of 2DNMs by hydrogen-bonding interactions to form biological scaffolds. Reprinted with permission from ref. [47], Copyright 2019, American Chemical Society. (**c**) Two-dimensional MOF material adsorbs serum protein as nano-scaffold. Reprinted with permission from ref. [48], Copyright 2022, American Chemical Society. (**d**) Ferritin-mimicking platinum nanoparticles’ complete modification of Ti_3_C_2_ nanosheets by cross-linking with GA. Reprinted with permission from ref. [49], Copyright 2024, MDPI.

The structural diversity of peptide side chains plays a pivotal role in facilitating the hybridization of peptides with various nanomaterials. In particular, aromatic amino acids such as tyrosine, phenylalanine, and tryptophan possess benzene ring structures in their side chains, enabling robust π–π stacking interactions with GO and other 2DNMs [43,44]. For instance, in the study by Liu et al., the biofunctionalization of GO was accomplished using peptide nanofibers (PNFs) formed from the KIIIIKYWYAF sequence. The aromatic residues tyrosine (Y) and phenylalanine (F) in the YWYAF motif engage in strong π–π stacking with the GO basal plane, driving both peptide self-assembly and stable adsorption onto GO. As illustrated in Figure 1a, these PNFs non-covalently coat the GO surface, creating a high-density scaffold of functional groups. This bio-templated interface not only promotes the in situ nucleation and growth of Au/Pt bimetallic nanoparticles via coordination with metal ions but also spatially organizes the resulting nanostructures, thereby enhancing nanoparticle dispersion and catalytic accessibility [45]. Similarly, Yang et al. employed PNFs as molecular bridges to introduce specific amino-functional sites for the conjugation of TiO_2_ onto g-C_3_N_4_ [46]. Through a freeze-drying process, TiO_2_-PNF-g-C_3_N_4_ hybrid aerogels were fabricated, which exhibited excellent photocatalytic degradation performance against pollutants such as methylene blue (MB) and rhodamine B (RhB). These composite materials demonstrate strong potential for applications in environmental remediation and bioanalytical detection [46].

In a related study, Li and colleagues engineered a series of peptides capable of self-assembling on the surfaces of various 2DNMs. As depicted in Figure 1b, the designed peptides featured glycine–alanine (GA) as a repeating sequence and were functionalized with different terminal amino acid residues to modulate their physicochemical properties. These peptides spontaneously formed stable β-sheet nanostructures through self-assembly processes and aligned linearly along the surfaces of 2DNMs such as GO and MoS_2_ in an ordered orientation. Studies have revealed that GA-repeat peptides exhibit enhanced stability due to their electrically neutral nature or reduced charge repulsion. Furthermore, longer peptide sequences demonstrate increased stability on 2DNMs, attributed to the formation of more extensive hydrogen-bonding networks. This research provides valuable insights into the design of biomolecular scaffolds for the surface functionalization of 2DNMs. Moreover, it highlights the potential of such peptide-based hybrid systems in expanding the biomedical applications of novel 2D composite materials [47].

### 2.2. Proteins

Proteins play a fundamental role in the biological processes of living organisms, serving as indispensable components of all cells and tissues. They are critically involved in a wide range of physiological functions, including molecular transport, immune defense, enzymatic catalysis, and hormonal regulation. In addition to their biological significance, proteins exhibit diverse nanostructural configurations that enable multifunctional applications, such as catalysis, targeted drug delivery, and fluorescence imaging [50,51,52]. The integration of proteins with 2DNMs significantly expands the functional landscape of these materials, enhancing their applicability across biomedical, diagnostic, and therapeutic domains.

When proteins are employed to modify 2DNMs, they can enhance the specificity of cellular targeting and serve as molecular recognition elements to facilitate the directed delivery of 2DNMs into specific cells or tissues. For instance, in a study conducted by Nagahama and collaborators, the adsorption, retention, and replenishment properties of 2D metal–organic frameworks (MOFs), particularly functionalized MIL-53(Al) (fMIL-53(Al)), were systematically evaluated for their application as a cellular scaffold for serum proteins. Utilizing a bottom–up fabrication strategy that combined solution infiltration with microwave irradiation, the researchers successfully synthesized fMIL-53(Al) thin-film scaffolds on polymer substrates with intrinsic cell adhesion capabilities (Figure 1c). The scaffolds’ design capitalized on the aromatic nature of the 1,4-benzenedicarboxylate ligands, which facilitated strong π–π stacking and hydrophobic interactions with aromatic and nonpolar amino acid residues in serum proteins. This non-covalent binding approach preserved the native tertiary structure of the proteins while enabling high-capacity adsorption. The porous architecture of the fMIL-53(Al) framework allowed for efficient protein loading, robust retention, and repeatable release cycles, supporting the sustained bioavailability of adsorbed proteins. Notably, proteins such as β-galactosidase retained significant biological activity upon adsorption, indicating that their structural integrity was largely maintained. Furthermore, the fMIL-53(Al) scaffolds supported long-term cell adhesion and proliferation, demonstrating their potential as bioactive interfaces. Protein retention studies showed that approximately 60% of the initially adsorbed proteins remained after extended incubation, underscoring the material’s stability and suitability for use as a long-term biomolecular interface [48].

In a separate study, Zhao et al. employed the positively charged protein Tamb to modify BP nanosheets through electrostatic interactions, resulting in the formation of a stable BP–Tamb 2D nanocomposite [53]. The Tamb protein effectively encapsulated the BP surface, creating a protective layer that significantly mitigated BP’s susceptibility to oxidation under ambient light and oxygen exposure. This surface modification not only enhanced the aqueous stability of BP but also preserved its intrinsic properties. Notably, the BP–Tamb composite exhibited excellent photothermal conversion efficiency when irradiated with a near-infrared (NIR) laser at a power density of 1 W/cm^2^. The preserved antigen-binding activity of the Tamb protein indicated that its tertiary structure remained intact, suggesting that no irreversible conformational changes occurred during photothermal excitation. This confirmed that the nanocomposite retained the inherent strong photothermal performance of BP and demonstrated promising antitumor efficacy in vitro, highlighting its potential for biomedical applications such as photothermal therapy [53]. Moreover, aberrant concentrations of specific proteins within biological systems are often associated with the onset of pathological conditions, making their sensitive and selective detection essential for early diagnosis and disease management. In this context, Mu et al. synthesized a hybrid protein–nanomaterial complex by encapsulating platinum nanoparticles within apoferritin (Pt@ApoF) [49], which was subsequently immobilized onto monolayer Ti_3_C_2_ (MXene) nanosheets through bioconjugation, forming the Pt@ApoF/Ti_3_C_2_ nanohybrid (Figure 1d). This composite was utilized to construct an electrochemical sensor with high selectivity and sensitivity toward nitrite ions, thereby demonstrating the effectiveness of the protein-assisted functionalization of 2DNMs for biosensing applications.

### 2.3. DNA/RNA

DNA and RNA, as nucleic acid polymers composed of repeating nucleotide units, serve essential roles in the storage and transmission of genetic information. Owing to their negatively charged phosphate backbones, they readily engage in electrostatic interactions with various molecules. Functionalizing 2DNMs with DNA or RNA enhances the targeting specificity and binding affinity of the nanomaterials, while the high surface area of 2DNMs concurrently facilitates an increased local concentration of nucleic acids. This reciprocal enhancement creates a synergistic effect that amplifies molecular recognition signals and improves detection sensitivity. Such integration of nucleic acids with 2DNMs holds significant promise for the development of advanced biosensing platforms and molecular diagnostics [54].

**Figure 2:**
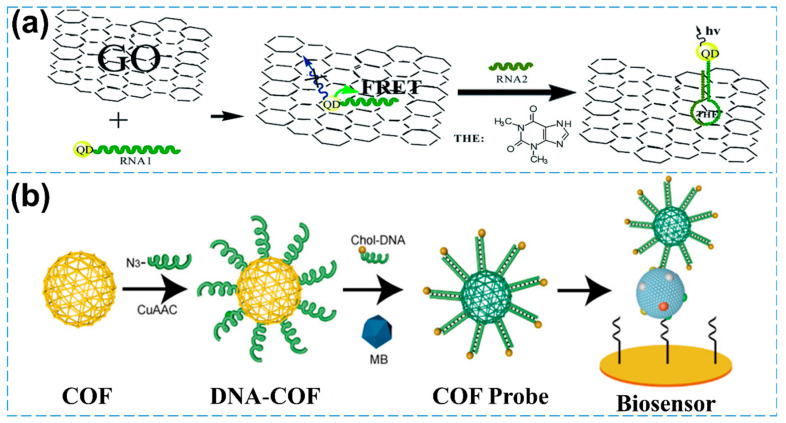
Modification of non-biological materials by DNA/RNA: (**a**) RNA-biomodified GO is used for fluorescence detection of target RNA. Reprinted with permission from ref. [55], Copyright 2019, Royal Society of Chemistry. (**b**) Nucleic acid functionalization of COF by DNA through covalent interactions. Reprinted with permission from ref. [56], Copyright 2022, American Chemical Society.

The cyclic aromatic structures of DNA and RNA nucleobases facilitate π–π stacking interactions with hexagonally arranged, layered 2DNMs, thereby enabling stable, non-covalent conjugation. Leveraging this interaction, Wang and colleagues functionalized RNA1 with fluorescent quantum dots and immobilized the construct on GO via π–π stacking (Figure 2a). The formed double-stranded RNA–theophylline complex (dsRNA–theophylline complex) enables highly selective detection by exploiting its differential affinity toward GO, thereby avoiding interference from non-target oligonucleotides. The inherent fluorescence-quenching properties of GO suppressed the quantum dot emission. However, upon the introduction of complementary target RNA2, preferential hybridization between RNA1 and RNA2 led to the displacement of the quantum dots from the GO surface, thereby restoring fluorescence and allowing for the sensitive detection of RNA2 [55]. In the work by Li et al., azide-modified DNA strands (N_3_-DNA) were covalently grafted onto an alkyne-rich covalent organic framework (COF) via a copper-catalyzed azide–alkyne cycloaddition (CuAAC) click reaction (Figure 2b), creating the first nucleic acid-functionalized COFs (DNA-COFs) [56]. Thanks to the bio-orthogonality of the CuAAC chemistry, the DNA retains its hybridization capability, while the exclusive reactivity between alkyne and azide groups ensures site-selective attachment on the COF surface. The resulting uniform DNA coating (i) provides a high density of accessible nucleic acid strands that markedly accelerates hybridization kinetics through multivalent base pairing and (ii) generates abundant negatively charged sites for the efficient electrostatic loading of molecular cargos. Importantly, because the click reaction proceeds under mild conditions without disrupting the COF’s porosity or framework integrity, DNA functionalization enhances the hybridization and cargo-loading properties of the COF without compromising its intrinsic physicochemical characteristics [56]. Beyond direct functionalization, nucleic acids can also serve as structural templates or molecular linkers between 2DNMs and other materials. For instance, Lee et al. synthesized silver nanoclusters (AgNCs) using DNA strands as templates and conjugated these complexes to GO via complementary DNA extensions. GO partially quenched the fluorescence of the DNA-AgNC complexes. Upon exposure to deoxyribonuclease I (DNase I), the enzyme specifically recognized and cleaved the exposed DNA strands, resulting in the detachment of the DNA-AgNC complexes from GO. This detachment reduced the quenching effect and led to a recovery of fluorescence, thus enabling the highly sensitive and selective detection of DNase I activity [57].

### 2.4. Bio-Enzymes

Bio-enzymes are catalytic biomolecules primarily composed of proteins, although a subset, termed ribozymes, are RNA-based and likewise exhibit catalytic activity. These macromolecular catalysts facilitate and accelerate biochemical reactions under physiologically mild conditions with remarkable efficiency, substrate specificity, and reaction selectivity. Due to their inherent biocompatibility and environmental benignity, bio-enzymes serve critical functions across a range of biological processes, including catalytic transformation, cellular differentiation, disease therapeutics, and biosensing applications.

**Figure 3:**
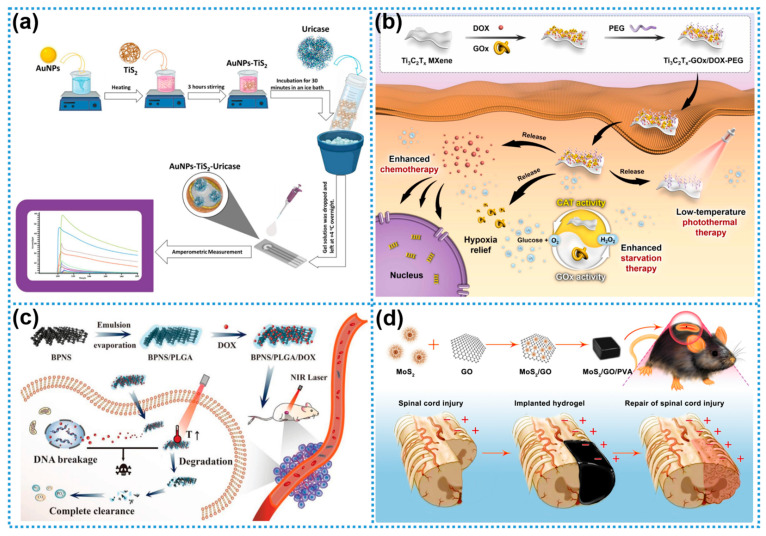
Modification of non-biological materials by bio-enzymes and biopolymers: (**a**) Gold nanoparticles and uricase-biomodified titanium disulfide for the construction of an electrochemical detection platform. Reprinted with permission from ref. [58], Copyright 2022, Elsevier. (**b**) Ti_3_C_2_T_x_-GOx/DOX-PEG enzyme system for antitumor therapy. Reprinted with permission from ref. [59], Copyright 2024, Elsevier. (**c**) PLGA-stabilized BP loaded with adriamycin mediating stable photothermal therapy. Reprinted with permission from ref. [62], Copyright 2020, Wiley-VCH. (**d**) MoS_2_ fused with PVA after GO modification by non-covalent modification to form MoS_2_/GO/PVA hydrogel. Reprinted with permission from ref. [63], Copyright 2022, BioMed Central.

Protein-based enzymes, composed of amino acid residues, are frequently employed in the biological functionalization of nanomaterials due to the structural diversity and tunability of their side chains. Their inherent substrate specificity and catalytic efficiency render them especially suitable for biosensor applications. For instance, Uygun and colleagues developed an enzymatic electrochemical sensor for the selective detection of uric acid, which demonstrated superior specificity compared to non-enzymatic counterparts. As illustrated in Figure 3a, titanium disulfide (TiS_2_), a representative transition metal dichalcogenide, was utilized as the sensing platform. Gold nanoparticles (AuNPs) and uricase were co-electrodeposited onto the TiS_2_ surface. The synergistic integration of AuNPs and uricase significantly enhanced the selectivity of the sensor, underscoring its potential utility in clinical diagnostic applications [58].

Despite their high catalytic efficiency and specificity, bio-enzymes face several limitations that hinder their broader application, including a narrow substrate range, sensitivity to environmental conditions, and high production costs. Integrating enzymes with 2DNMs offers a promising strategy to enhance their catalytic performance, stability, and reusability while simultaneously imparting additional functionalities to the nanomaterials. Qiao et al. engineered a Ti_3_C_2_ MXene-based platform exhibiting intrinsic catalase-mimetic activity, which demonstrated potent therapeutic efficacy in both in vitro and in vivo antitumor models [59]. The MXene surface was modified with silane coupling agents to introduce abundant amino groups and positive surface charges. As depicted in Figure 3b, the positively charged Ti_3_C_2_ MXene engaged in electrostatic interactions with the negatively charged glucose oxidase (GOx), while Schiff base (–C=N–) linkages were formed between surface amino groups and aldehyde functionalities of doxorubicin (DOX). Further PEGylation yielded the Ti_3_C_2_T_x_-GOx/DOX-PEG enzyme–nanomaterial hybrid [59]. This multifunctional system leveraged the photothermal properties of MXene and enzymatic cascade reactions to mitigate tumor hypoxia, thereby enhancing the efficacy of photothermal therapy. In a separate study, Krukiewicz et al. developed GO/poly(methyl methacrylate) (GO/PMMA) composites, which were subsequently biofunctionalized with alkaline phosphatase (ALP) [60]. The resulting hybrid materials significantly promoted osteogenic differentiation. Compared to traditional single-component repair strategies, the ALP-functionalized GO/PMMA composites exhibited superior bone regeneration performance, demonstrating their potential in tissue engineering applications [60]. Wang et al. successfully constructed a novel biomimetic multienzyme nano-reactor (GOx@MAF-7(Fe), abbreviated as GMF) by encapsulating GOx within a metal–azolate framework MAF-7(Fe) through a one-step encapsulation strategy [61]. Owing to the excellent hydrophilicity, biocompatibility, and porous structure of MAF-7(Fe), the platform significantly enhanced the stability, thermal resistance, and long-term storage capability of GOx while also achieving efficient enzyme immobilization. However, despite the promising results from enzyme–2DNM composites, biocompatibility and biosafety remain enduring and critical issues in their in vivo applications. Conducting systematic and comprehensive long-term biosafety assessments for these enzyme–2DNM composites is indispensable—not only to validate their clinical feasibility but also to lay the groundwork for their successful translation into practical medical therapies [61].

### 2.5. Biopolymers

Biopolymers are highly esteemed in biomedical and environmental applications due to their exceptional biocompatibility and biodegradability under stimuli such as microbial activity, temperature, and humidity. Representative examples include polyvinyl alcohol (PVA), polyglycolic acid (PGA), and their copolymers, notably poly(lactic-co-glycolic acid) (PLGA). When employed for the functionalization of 2DNMs, biopolymers not only improve their biocompatibility but also introduce beneficial properties such as controlled degradation and programmable drug release.

The intrinsic capacity of biopolymers to encapsulate therapeutic agents during polymerization and facilitate their controlled release upon degradation makes them ideal candidates for 2DNM surface engineering. Biopolymer–2DNM composites can employ programmable degradation to stay in sync with tissue-healing timelines [64]. Besides passive degradation via hydrolysis or enzymatic cleavage, pH-, temperature-, light-, and electric/magnetic field-responsive functional groups can be grafted onto the surfaces of 2DNMs (e.g., GO, MXene) or incorporated into smart cross-linkers to trigger on-demand drug release [65,66]. By tuning the polymer composition, cross-linking density, and processing conditions, the mechanical strength and degradation kinetics of coatings or hydrogels become highly adjustable, enabling the precise matching of the target tissue’s stiffness and remodeling rate [67]. Xu et al. demonstrated this potential by modifying black phosphorus nanosheets (BPNSs) with PLGA to overcome their inherent aqueous instability while maintaining their photothermal and biodegradable properties [62]. As illustrated in Figure 3c, PLGA formed a protective shell around the BPNSs, improving their structural integrity and enabling the electrostatic loading of DOX onto the nanosheet surface. This encapsulation markedly reduced the degradation rate of BPNSs during photothermal treatment, thereby extending their therapeutic window. Since the PLGA coating itself does not absorb or convert near-infrared light, the photothermal conversion efficiency of the BPNS/PLGA composite was calculated to be 32.7%, and the concentration of BPNS/PLGA affects its photothermal performance. Moreover, the co-loaded DOX synergistically enhanced the system’s antitumor activity—suppressing tumor recurrence—and the PLGA shell facilitated the simultaneous delivery of additional chemotherapeutics, enabling combinatorial chemo-photothermal therapy with superior efficacy [62].

**Figure 4:**
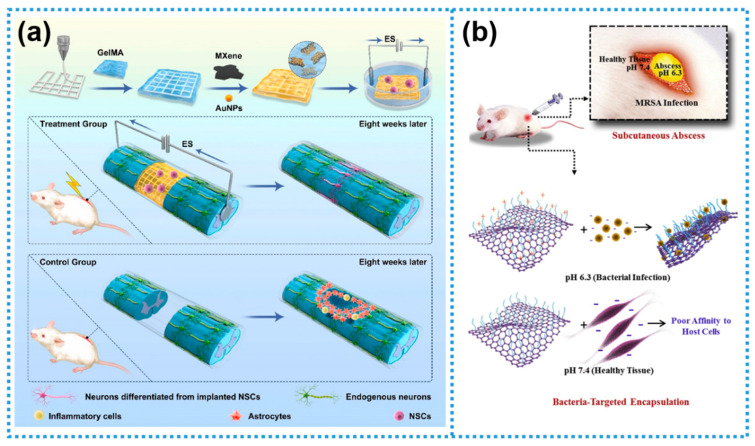
Modification of non-biological materials by biopolymers and bioactive polysaccharides: (**a**) MXene-based gelatin hydrogel (MAu-GelMA) promotes nerve regeneration and motor recovery after SCI in rats. Reprinted with permission from ref. [68], Copyright 2023, Elsevier. (**b**) Ethylene glycol chitosan-functionalized carboxylic graphene (GCS-CG) for targeted antibacterial application. Reprinted with permission from ref. [69], Copyright 2018, Elsevier.

In another study, Chen and co-workers developed a multifunctional hydrogel by integrating PVA with GO and MoS_2_ through repeated freeze–thaw cycles, yielding MoS_2_/GO/PVA hydrogels. As shown in Figure 3d, the inclusion of MoS_2_ improved GO’s mechanical flexibility and electrical conductivity, while the inherent anti-inflammatory properties of PVA promoted tissue regeneration. This hydrogel significantly enhanced cell differentiation and tissue repair in vivo, highlighting its potential for spinal cord injury repair and wound-healing applications [63]. Separately, Kong et al. engineered a degradable, electroactive hydrogel (MAu-GelMA) with a hierarchically biomimetic structure by synergistically combining biopolymers and nanomaterials [68]. As depicted in Figure 4a, the MXene-based gelatin methacrylate hydrogel exhibited excellent conductivity, facilitating efficient electrical signal transduction. When paired with electrical stimulation (ES) therapy, this platform significantly promoted nerve regeneration and motor function recovery in spinal cord injury (SCI) models, underscoring the therapeutic promise of biopolymer–nanomaterial hybrid systems [68].

### 2.6. Bioactive Polysaccharides

Bioactive polysaccharides are widely distributed natural macromolecules that serve as structural components in the extracellular matrices of animals and the cell walls of plants and microorganisms, where they contribute to a range of physiological functions, including immune modulation, cell signaling, and tissue regeneration [70]. These natural macromolecules have garnered significant attention for their therapeutic potential, particularly in oncology. Polysaccharides such as chitosan, alginate, hyaluronic acid (HA), and dextran are characterized by their biocompatibility, biodegradability, and immunomodulatory capabilities. Extensive research has documented their antimicrobial activity, ability to adsorb heavy metals, and utility in disease treatment and tissue repair applications [71,72]. The inherent biodegradability of polysaccharide-based nanocomposites contributes to their safe clearance in vivo by enzymatic or hydrolytic degradation into non-toxic byproducts, reducing long-term accumulation and improving biosafety [73].

In the context of cancer therapy, bioactive polysaccharides primarily exert their effects through two distinct mechanisms: acting as immunomodulators to enhance host immune responses or directly targeting pathological cells for elimination. When employed in conjunction with 2DNMs, surface functionalization with these polysaccharides enables multimodal therapeutic approaches, such as the integration of targeted drug delivery and photothermal therapy. Their molecular structures are rich in functional groups such as hydroxyl, amino, and carboxyl moieties which facilitate a range of interactions with 2DNMs, including hydrogen bonding, electrostatic interactions, and covalent conjugation.

Chitosan, a naturally occurring cationic polysaccharide, exemplifies this versatility. Due to its positive charge, chitosan can form stable electrostatic complexes with negatively charged materials such as GO, thereby enhancing GO’s dispersion and colloidal stability in aqueous environments. Qian et al. developed a glycol chitosan-functionalized, carboxylated GO composite (GCS-CG) to target bacterial infections in acidic abscess environments [69]. Glycol chitosan (GCS), a water-soluble derivative of chitosan, exhibits pH-responsive charge-switching behavior. As illustrated in Figure 4b, GCS-CG transitions from a net negative to positive charge in acidic microenvironments, enabling it to selectively interact with the negatively charged membranes of pathogenic bacteria. This electrostatic attraction facilitates bacterial encapsulation. Upon near-infrared (NIR) light exposure, the GCS-CG nanocomposite efficiently converts photonic energy into heat, achieving localized photothermal ablation of the encapsulated bacteria. Importantly, this pH-dependent behavior ensures minimal off-target effects, preserving surrounding healthy tissues [69].

### 2.7. Biomolecule Nanointerface Engineering

Interfacial engineering between biomolecules and 2DNMs has emerged as a cutting-edge domain within biomedical nanotechnology. At its core, this discipline seeks to achieve the synergistic enhancement of both biological functionality and the intrinsic physicochemical properties of 2DNMs through precise molecular-level design and dynamic interfacial modulation [74]. The overarching goal is to develop interfacial platforms that retain the superior characteristics of 2DNMs such as high surface area, conductivity, and mechanical strength while simultaneously harnessing the molecular specificity and functional diversity of biomolecules. Due to their excellent biocompatibility, structural stability, and functional versatility, biomolecules including peptides, proteins, nucleic acids (DNA/RNA), bio-enzymes, biopolymers, and bioactive polysaccharides can be effectively immobilized onto 2DNM surfaces, enabling the creation of advanced bio–nanohybrid systems with tailored biomedical applications [75].

**Figure 5:**
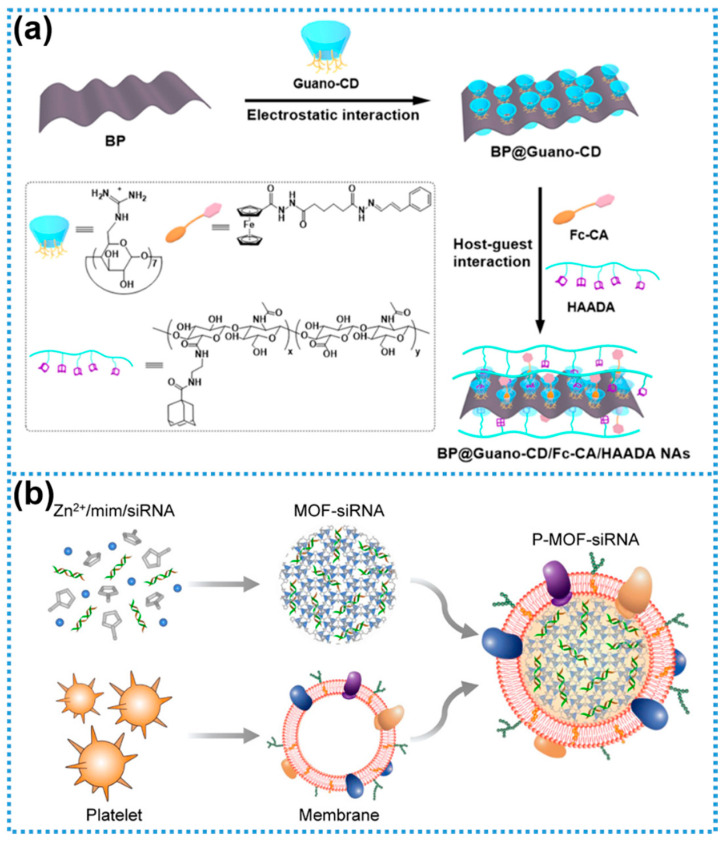
Interfacial engineering of biomolecules with 2DNMs: (**a**) Construction of multifunctional supramolecular assemblies based on BP nanosheets using Guano-CD, Fc-CA, and HAADA. Reprinted with permission from ref. [77], Copyright 2025, Elsevier. (**b**) MOF-based targeted gene delivery system (P-MOF-siRNA). Reprinted with permission from ref. [80], Copyright 2020, American Association for the Advancement of Science.

Biomimetic interface engineering offers a powerful platform for the rational design of multifunctional nanomaterials, providing both structural versatility and functional specificity essential for applications in high-sensitivity biosensing, targeted therapeutics, and tissue regeneration. This approach also establishes a critical technological foundation for the continued development of nanomedicine [76]. In a representative study by Chen and colleagues [77], a multifunctional supramolecular assembly (BP@Guano-CD/Fc-CA/HAADA) was constructed using BP nanosheets as the core material through a biomimetic interface engineering strategy. This system ingeniously integrates electrostatic self-assembly with dynamic host–guest recognition. As illustrated in Figure 5a, positively charged guanidyl-functionalized β-cyclodextrin (Guano-CD) was first electrostatically adsorbed onto the negatively charged BP nanosheets. Subsequently, ferrocene-modified cinnamaldehyde (Fc-CA) was encapsulated within the Guano-CD cavity via host–guest interactions. Simultaneously, adamantane-modified hyaluronic acid (HA-ADA) was selectively bound to the β-cyclodextrin units, yielding a highly integrated, multifunctional BP-based supramolecular complex that exhibits excellent stability, biocompatibility, and cancer-cell-targeting capabilities.

Exploiting the synergistic interactions at the bio–nano interface, Zhang et al. developed a biomimetic bone matrix nanofiber scaffold via electrospinning, utilizing a composite of polycaprolactone (PCL), collagen, and nano-hydroxyapatite [78]. This nanofibrous matrix emulates the structural and compositional characteristics of native bone extracellular matrix and provides valuable insights for the rational design of next-generation scaffolds for bone tissue engineering applications [78]. In a separate study, Song and colleagues functionalized GO with the cell-adhesive peptide sequence arginine–glycine–aspartic acid (RGD) using carbodiimide chemistry (EDC/NHS-mediated coupling), effectively forming a bioactive RGD-GO 2D composite interface. This functionalization enhanced the biocompatibility and cellular adhesion properties of the material, making it suitable for biological applications [79]. Additionally, Zhuang et al. engineered a novel gene delivery platform based on a MOF, as illustrated in Figure 5b [80]. Using biocompatible zeolitic imidazolate framework-8 (ZIF-8) as the carrier, small interfering RNA (siRNA) was incorporated into the MOF matrix through coordination-driven self-assembly with zinc ions and 2-methylimidazole. Subsequent surface functionalization with platelet-derived membranes conferred active targeting capabilities, yielding a biomimetic siRNA delivery system (P-MOF-siRNA) with promising therapeutic potential [80].

### 2.8. Summary of Biomodification

In order to assist in the understanding of the above presentation and discussion, we summarize common biomaterials used for the modification of 2DNMs with their modification strategies, as shown in Table 1. The content is categorized and summarized in terms of two approaches: covalent interactions and non-covalent interactions.

## 3. Biomimetic Synthesis of 2DNMs

The biomimetic synthesis of 2DNMs is inspired by the principle of “learning from nature”, wherein the structural formation processes of natural organisms are emulated to engineer materials with unique functionalities under mild, environmentally benign conditions [81]. Key biomimetic strategies employed in the synthesis of 2DNMs include bio-templating, molecularly directed self-assembly, biomineralization, and dynamic biomimetic interface engineering. Structural biomimicry emphasizes energy-efficient fabrication, typically requiring low energy input and offering advantages such as synthesis simplicity, rapid production, and enhanced safety. This approach effectively addresses the limitations associated with conventional synthesis methods, particularly in terms of energy consumption, environmental sustainability, and morphological control. In contrast, functional biomimicry focuses on the rational modification of 2DNMs to emulate specific biological behaviors, thereby imparting intelligent responsiveness, enhanced biocompatibility, and superior functional performance. Collectively, biomimetic synthesis strategies enable the environmentally sustainable, structurally precise, and functionally integrated fabrication of 2DNMs. Consequently, the development and optimization of biomimetic methodologies hold significant promise for advancing the design and application scope of 2DNM-based hybrid systems across diverse scientific and biomedical domains.

### 3.1. Bio-Templating

The use of bioactive substances as templates represents a widely employed and effective strategy in the biomimetic synthesis of 2DNMs. Biomolecules such as peptides, proteins, nucleic acids (DNA/RNA), and biomass-derived polysaccharides not only serve as structural templates during material synthesis but also function as functional modifiers of 2DNMs. This dual role enables precise morphological control and facilitates the integration of diverse functionalities, thereby achieving customizable and multifunctional nanomaterial systems [82,83,84,85].

**Figure 6:**
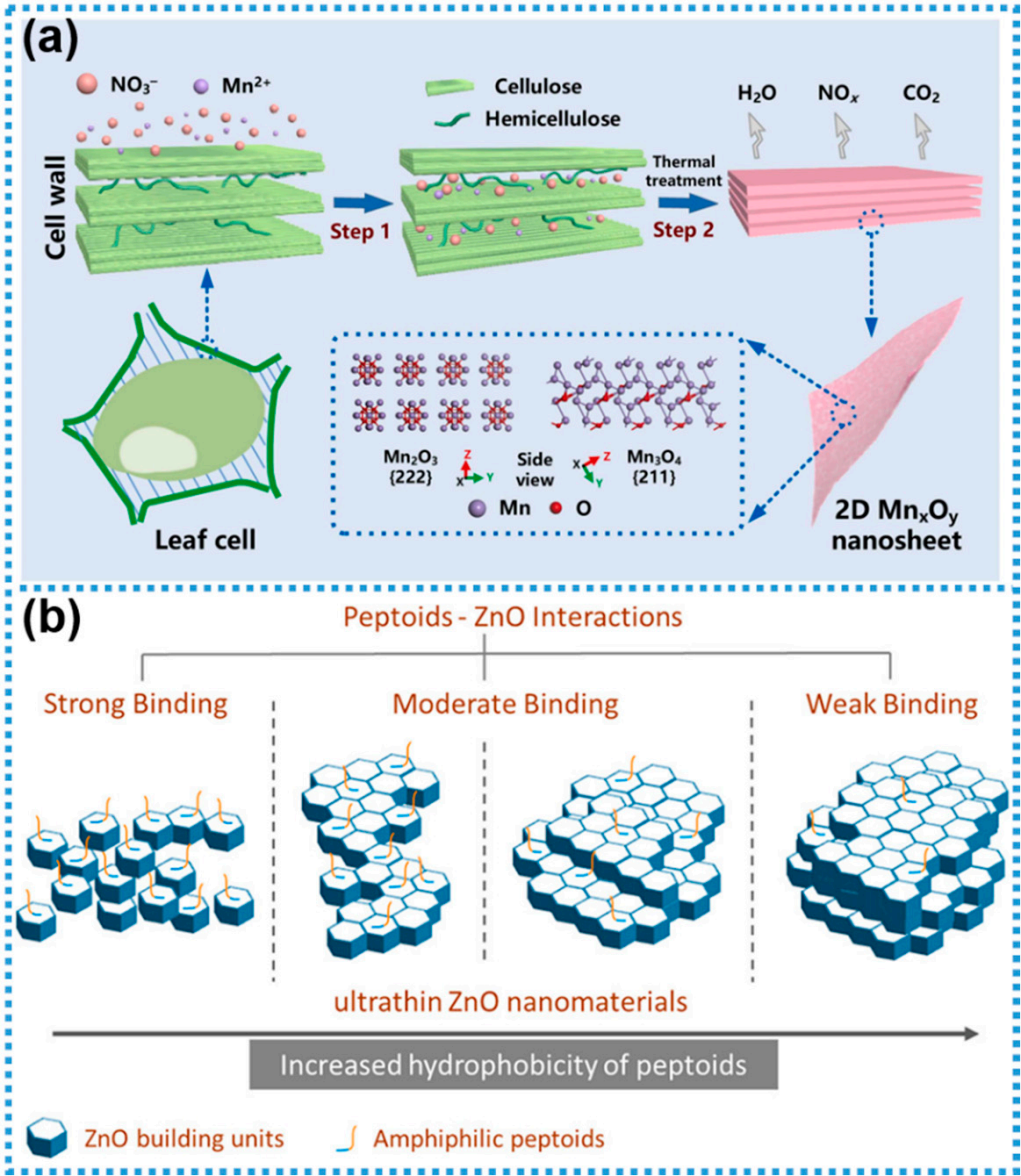
Biomimetic synthesis of non-biological materials using biologically active substances as templates: (**a**) 2D manganese oxide nanosheets using cabbage leaf cells as a template. Reprinted with permission from ref. [87], Copyright 2024, Elsevier. (**b**) Biomimetic synthesis of 2D zinc oxide nanomaterials using peptides as a template. Reprinted with permission from ref. [88], Copyright 2023, American Chemical Society.

The unique “template modification” synergy offered by bioactive substances plays a pivotal role in both the structural engineering and functionalization of 2DNMs. This approach allows for the accurate replication of biomimetic architectures while concurrently introducing intelligent responsiveness through molecular recognition and selective interaction mechanisms. For instance, Xiao and colleagues employed collagen as a biomolecular template to guide the nucleation of manganese phosphate, resulting in the formation of uniform protein–manganese phosphate nanoflowers. These nanostructures exhibited high porosity, favorable biocompatibility, and enhanced functional performance [86]. In a separate study, Wu et al. utilized cabbage leaf cells as biomimetic templates to synthesize 2D manganese oxide nanosheets through a bottom–up fabrication process [87]. As illustrated in Figure 6a, an osmotic pressure gradient between intracellular and extracellular environments facilitated the diffusion of manganese ions into the layered cellulose matrix. These ions subsequently coordinated with organic functional groups in the plant cell walls. Following high-temperature pyrolysis, the biological template was removed, and confined nucleation within the cellulose matrix gave rise to well-defined 2D manganese oxide nanosheets. Additionally, Yang and co-workers demonstrated the use of hydrophobic peptides as morphology-directing agents in the synthesis of 2D ZnO nanostructures [88]. By increasing the number of hydrophobic peptide chains, the interaction with ZnO precursors was strengthened, reducing crystallization interference and ultimately yielding highly uniform 2D ZnO nanosheets (Figure 6b).

### 3.2. Biomolecule-Directed Self-Assembly

Biomolecule-directed self-assembly represents a prominent strategy in biomimetic synthesis, wherein synthetic or naturally occurring biomolecules including peptides, proteins, nucleic acids (DNA/RNA), and polysaccharides spontaneously organize into well-defined nanostructures through non-covalent interactions such as hydrogen bonding, electrostatic interactions, π–π stacking, and van der Waals forces [40].

**Figure 7:**
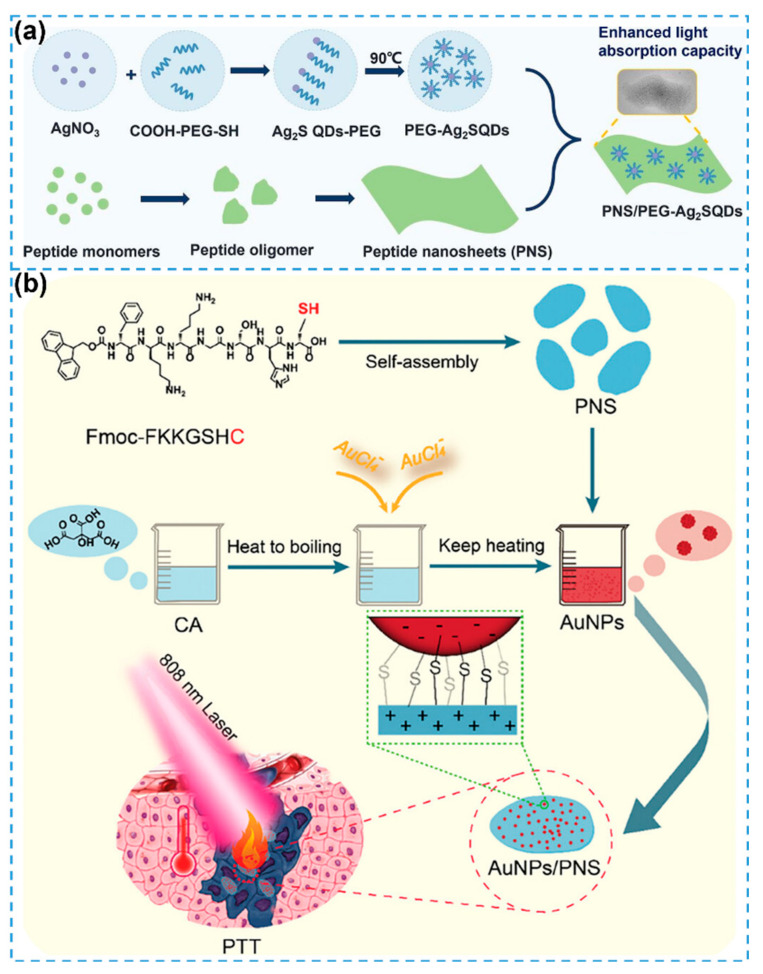
Biomimetic synthesis of non-biological materials by biomolecule-directed self-assembly: (**a**) Schematic synthesis of PNS/PEG-Ag_2_SQDs 2D multifunctional nanoplatforms. Reprinted with permission from ref. [92], Copyright 2024, Elsevier. (**b**) PNS-encapsulated gold nanoparticles for synthesis of 2D inorganic/organic nanohybrid materials, AuNPs/PNS. Reprinted with permission from ref. [93], Copyright 2024, Wiley-VCH.

Common approaches within this framework include the regulated synthesis of peptides/proteins and DNA/RNA-mediated assembly processes, which enable precise structural control and functional integration at the nanoscale [89,90]. Nanomaterials constructed through biomolecule-directed self-assembly inherently exhibit superior biocompatibility and bioactivity, significantly broadening the scope for the development of advanced biomaterials and multifunctional platforms for biomedical applications [91]. For instance, Luan and colleagues designed a biocompatible nanohybrid system (2D PNS/PEG-Ag_2_S QDs) capable of simultaneous bioimaging and photothermal therapy (PTT) for cancer treatment. As illustrated in Figure 7a, a synthetic peptide bearing the Fmoc-FKKGSH sequence underwent self-assembly into uniform 2D peptide nanosheets (PNSs), offering an abundance of active binding sites. These PNSs served as a structural scaffold for the immobilization of PEG-modified Ag_2_S quantum dots, thereby facilitating the one-step formation of a multifunctional 2D nanoplatform. This hybrid system demonstrated promising performance in synergistic therapeutic and diagnostic applications [92].

In a related study, Xu and colleagues designed an amphiphilic peptide with the sequence Fmoc-FKKGSHC, which self-assembled into 2D PNSs under controlled conditions. These PNSs effectively encapsulated gold nanoparticles (AuNPs), resulting in a biomimetic 2D organic/inorganic nanohybrid (AuNPs/PNS), as illustrated in Figure 7b [93]. Similarly, our group synthesized bimetallic CuCoO_2_ nanosheets and employed a peptide with the sequence KIIIIKYWYAF to form PNFs through self-assembly. The resulting nanohybrid (BMNS-PNF), formed via non-covalent interactions between the bimetallic nanosheets and the PNFs, exhibited excellent biocompatibility and robust photothermal therapeutic performance [94]. Additionally, Liu and colleagues leveraged the structural programmability of DNA origami to construct sophisticated 2D nanostructures. By precisely adjusting the positions of cholesterol-modified DNA strands and introducing hydrophobic interactions, they achieved the controlled folding of planar DNA sheets into complex, programmable architectures. This approach significantly expands the capabilities of DNA nanotechnology, offering new avenues for diverse biomedical and nanotechnological applications [95].

### 3.3. Biomineralization Strategies

Biomineralization-based synthesis represents a widely utilized biomimetic strategy that emulates the natural formation processes of biological minerals, such as seashells and bones, to enable the controlled synthesis of 2D inorganic nanomaterials via organic molecular regulation [96,97]. This biomimetic approach typically progresses through four distinct stages: (i) the self-assembly of biomacromolecules into well-ordered supramolecular structures; (ii) the selective adsorption of inorganic ions facilitated by functional groups at molecular interfaces, which promotes site-specific crystal nucleation; (iii) crystal growth regulation through facet-selective mechanisms to produce uniform subunits; and (iv) the hierarchical, cross-scale organization of these subunits into macroscopic functional materials with tailored properties [98].

**Figure 8:**
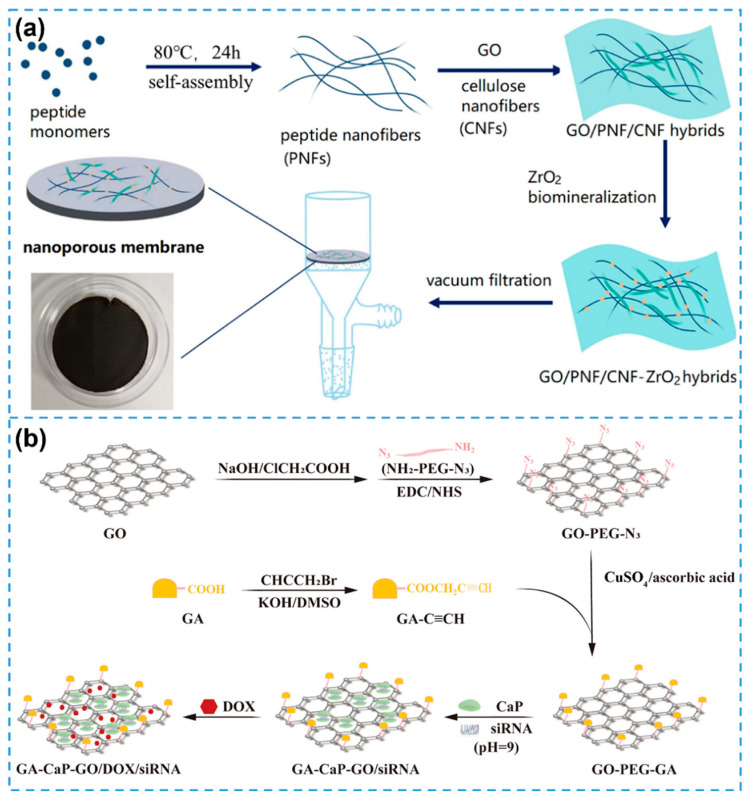
Biomimetic synthesis of abiotic materials by biomineralization: (**a**) Biomimetic synergistic synthesis of zirconium dioxide nanoparticles by biomimetic mineralization of peptide and cellulose nanofibers on GO surface. Reprinted with permission from ref. [100], Copyright 2022, Elsevier. (**b**) Construction of biomineralized 2DNMs for drug delivery using GO as a template. Reprinted with permission from ref. [101], Copyright 2024, Springer Netherlands.

The organic/inorganic co-assembly strategy rooted in biomineralization presents a novel paradigm for achieving cross-scale precision in material fabrication while simultaneously offering a universal theoretical framework for the rational design of advanced biomimetic smart materials [99]. For instance, Chen and colleagues harnessed the biomimetic synergy between PNFs and cellulose nanofibers (CNFs) to facilitate the controlled biomineralization of zirconium dioxide (ZrO_2_) nanoparticles [100]. As illustrated in Figure 8a, a ternary hybrid substrate (GO/PNF/CNF) was engineered through a combination of non-covalent intermolecular interactions among GO nanosheets, PNFs, and CNFs. This hybrid matrix served as both an adsorption platform and a nucleation template for ZrO_2_ nanoparticle formation. By finely tuning the solution pH to simulate a biomimetic microenvironment, the directional growth of highly dispersed ZrO_2_ nanoparticles was achieved at the organic/inorganic interface, culminating in the formation of a hierarchically organized GO/PNF/CNF-ZrO_2_ composite with mineralized structural features and enhanced functional properties.

In the study conducted by Kim et al. [102], a biomimetic composite nanofiber scaffold was developed through the integration of albumin-assisted liquid-phase exfoliation of 2D MoS_2_ nanosheets with electrospinning techniques. This process yielded a mineralized PZM (polymer/zirconium–albumin/MoS_2_) composite scaffold, characterized by enhanced functionality and structural mimicry of natural extracellular matrices. In a separate investigation, Cheng et al. designed a GO-templated intelligent drug delivery system (GA-CaP-GO/DOX/siRNA), inspired by biomineralization mechanisms inherent to 2DNMs [101]. As illustrated in Figure 8b, the researchers initially constructed a biomimetic interface on GO surfaces by grafting PEG and conjugating the targeting ligand GA via click chemistry, forming GO-PEG-GA nanosheets with both biological stability and tumor-targeting capability. Utilizing the biomineralization kinetics of calcium phosphate (CaP), the co-deposition of CaP and siRNA was subsequently performed on the modified GO surface. DOX was then loaded onto the nanosheets through π–π stacking interactions, resulting in a dual-drug-loaded, GO-based biomineralized nanoplatform with potential for synergistic cancer therapy [101].

### 3.4. Biomimetic Functional Integration

In the context of functional biomimicry, the integration of bioinspired properties into 2DNMs represents a pivotal direction for advancing material design. Emulating natural structures and phenomena such as the superhydrophobic surfaces of lotus leaves, the dynamic color adaptation of chameleon skin, the optical features of butterfly wing scales, and the selective permeability of cellular membranes enables 2DNMs to exhibit intelligent responsiveness, enhanced biocompatibility, and multifunctional capabilities. As research in this domain continues to evolve, an increasing number of biomimetic materials have been developed that not only replicate biological functions in vitro but also demonstrate promising performance in vivo, thereby broadening their applicability in biomedical and environmental fields [103].

**Figure 9:**
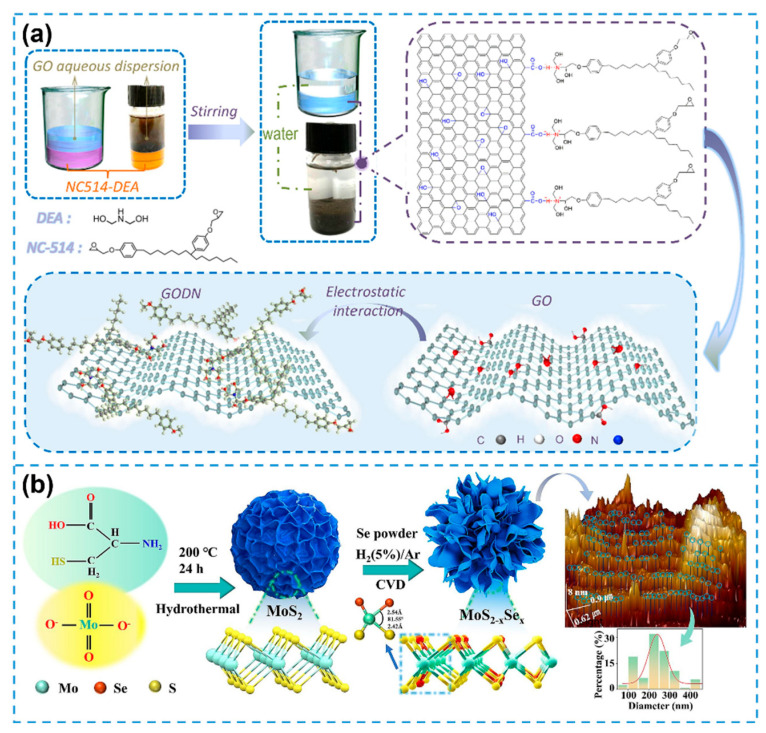
Biomimetic functional integration of 2DNMs: (**a**) Synthesis of bio-based nanocomposites based on GO via non-covalent interactions: biomimetic synthesis of DN with GO as a matrix. Reprinted with permission from ref. [105], Copyright 2021, Elsevier. (**b**) Schematic of the preparation of MoS_2−x_Se_x_ alloy nanocomposites characterized by the micro- and nanostructure of the lotus leaf surface. Reprinted with permission from ref. [107], Copyright 2024, Elsevier.

Certain 2DNMs, such as GO and BP, have demonstrated inherent bioactivity in promoting cellular differentiation, thus providing a solid foundation for their application in biomimetic systems. For instance, Guo et al. introduced a biomimetic photocatalytic 2DNM hemin–bismuth tungstate (HBW), in which hemin molecules were anchored onto bismuth tungstate (BWO) via a straightforward hydrothermal synthesis [104]. The incorporation of hemin notably enhanced the electron transport capabilities of the composite, thereby improving the photocatalytic efficiency of BWO. Meanwhile, the BWO matrix effectively dispersed the hemin molecules, preventing their aggregation into catalytically inactive dimers and maintaining optimal performance. The resulting HBW nanomaterial successfully retained the photocatalytic activity of hemin along with the favorable electronic properties of BWO, establishing a promising platform for biomimetic photocatalysis. In a separate study, Zhao et al. proposed a sustainable synthesis route for bio-based nanocomposites [105]. As illustrated in Figure 9a, cardanol derived from renewable cashew nut shell liquid was utilized as the primary raw material. Through nucleophilic ring-opening reactions, diethanolamine (DEA) was grafted onto the cardanol epoxy resin backbone to produce a modified epoxy resin (DN). This DN matrix was subsequently integrated with GO via electrostatic interactions, yielding a renewable and functional nanocomposite material.

Inspired by natural wood structures, Chen et al. engineered biomimetic wood-like coaxial fibers composed of MXene encapsulated within GO shells [106]. By exploiting the excellent gelation behavior and mechanical robustness of GO, the MXene cores were uniformly and tightly enclosed, resulting in coaxial fiber architectures that combine high electrical conductivity with enhanced mechanical durability. The protective GO sheath not only improved thermal stability but also minimized the degradation of MXene under elevated temperatures, highlighting the material’s promise for applications in flexible and wearable electronics. Similarly, inspired by the superhydrophobic characteristics of lotus leaf surfaces, Shi and colleagues designed MoS_2−x_Se_x_ alloy nanocomposites exhibiting hierarchical micro-/nanostructures analogous to those found in lotus leaves [107]. As illustrated in Figure 9b, molybdate ions were reduced by the –SH groups of L-cysteine during a hydrothermal process, guiding the directional assembly of layered MoS_2_ precursors. Subsequent chemical vapor deposition (CVD) enabled the formation of MoS_2−x_Se_x_ alloy nanosheets with a tunable composition and surface morphology. This structural mimicry conferred enhanced hydrophobicity and significantly improved gas-sensing performance through synergistic functional optimization.

### 3.5. Summary of Biomimetic Synthesis

To facilitate a clearer understanding of the aforementioned concepts and discussions, a comparative overview of the four principal strategies utilized in the biomimetic synthesis of 2DNMs is provided in Table 2. This table systematically summarizes the representative templates or mediating agents involved in each approach, along with the corresponding materials synthesized or their functional attributes.

## 4. Biosensor Applications of 2DNMs

Conventional analytical techniques such as ultraviolet–visible (UV-Vis) spectroscopy, high-performance liquid chromatography (HPLC), liquid chromatography–mass spectrometry (LC-MS), and atomic absorption spectroscopy (AAS) have been extensively utilized for the quantitative and qualitative detection of a wide range of analytes, including those present at ultra-trace levels. Despite their sensitivity and accuracy, these methods often necessitate labor-intensive sample preparation, complex operational procedures, and rigorous environmental control for both instrument maintenance and analytical performance. Moreover, their applicability is notably constrained in the detection of transient or biologically relevant targets such as reactive oxygen species (ROS), viruses, hormones, and biomolecules.

In contrast, biosensors offer significant advantages, including high specificity, excellent selectivity, rapid detection, and the potential for real-time and visualizable outputs. Based on their detection mechanisms, biosensors are broadly classified into electrochemical, colorimetric, fluorescent, photoelectric, and multimodal types. The integration of 2DNMs into biosensor platforms has garnered substantial interest due to their unique physicochemical properties, large surface-to-volume ratio, and facile surface functionalization. These attributes significantly enhance biosensor performance, enabling sensitive and selective detection across a wide spectrum of biological and chemical targets [108,109]. Recent studies have shown that integrating two-dimensional nanomaterial-based biosensors with portable and wearable platforms enables the real-time in vivo monitoring of metabolites, proteins, and other biomarkers, thereby overcoming the constraints of laboratory-bound settings and facilitating continuous health monitoring [110,111].

### 4.1. Colorimetric Biosensors

Compared to conventional analytical techniques, colorimetric biosensors offer distinct advantages including visual readout capability, rapid response, low operational cost, and minimal instrumentation requirements. These characteristics make them particularly suitable for on-site and point-of-care diagnostics. Owing to their inherent molecular recognition specificity, colorimetric biosensors enable the accurate and reliable detection of target analytes even within complex biological or environmental matrices. Furthermore, their compatibility with paper-based platforms facilitates the development of portable and user-friendly diagnostic tools for field applications. To endow these systems with both visual signal generation and high specificity, functional nanomaterials are frequently incorporated into the sensor design. Noble metal nanoparticles such as gold (Au), silver (Ag), and platinum (Pt) are widely employed due to their excellent catalytic activity, which significantly enhances the rate and efficiency of chromogenic reactions. The choice of chromogenic substrates is critical for signal output, relying on distinct color transitions associated with redox reactions in solution. Commonly utilized substrates include 3,3′,5,5′-tetramethylbenzidine (TMB), diaminobenzidine (DAB), and 2,2′-azino-bis(3-ethylbenzothiazoline-6-sulfonic acid) (ABTS), each of which enables sensitive visual detection by undergoing pronounced colorimetric shifts during electron transfer processes. These features collectively underpin the broad applicability of colorimetric biosensors for the detection of DNA, proteins, viruses, small molecules, and metal ions [112,113].

**Figure 10:**
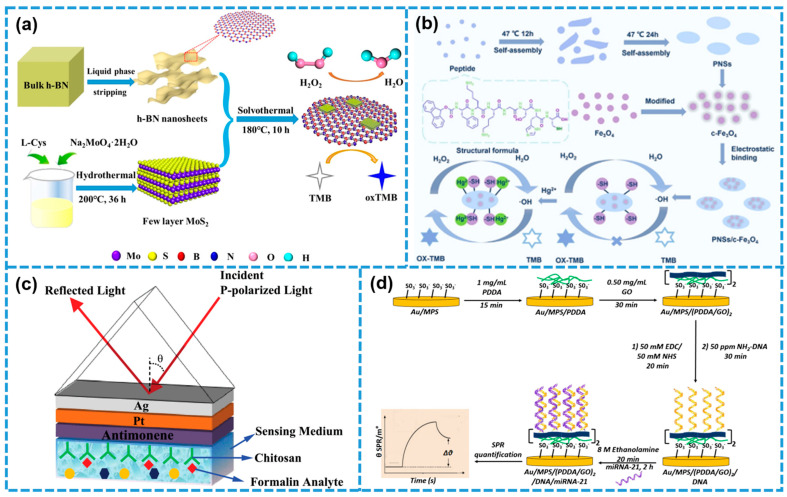
Two-dimensional nanomaterials in colorimetric and SPR biosensors: (**a**) h-BN/N-MoS_2_ 2D nanohybrid materials with enhanced peroxidase activity to construct occlusion biosensors for H_2_O_2_ detection. Reprinted with permission from ref. [120], Copyright 2020, Wiley-VCH. (**b**) c-Fe_3_O_4_ MNPs functionalized for PNS for colorimetric detection of Hg^2+^. Reprinted with permission from ref. [121], Copyright 2023, Royal Society of Chemistry. (**c**) Chitosan-modified 2D antimony alkene nanomaterial SPR biosensor for detection of formalin. Reprinted with permission from ref. [122], Copyright 2022, IEEE. (**d**) DNA-biomodified GO for SPR analysis to detect miRNA-21. Reprinted with permission from ref. [123], Copyright 2020, Springer Netherlands.

In colorimetric assays, complex biological matrices (such as serum or saliva) contain abundant proteins, enzymes, and other substances that can interfere with the optical signal [114,115]. To reduce background interference and enhance detection specificity, multiple strategies are typically employed. These include using centrifugation or microporous filtration to remove macromolecules and particulate impurities; applying surface modifications (for example, antibody coating, molecularly imprinted polymers, or PEG blocking) to suppress nonspecific adsorption; and incorporating internal standard correction, ratiometric signal readouts, or nanozyme “on–off” catalytic systems in the material design and assay scheme [116,117]. Together, these measures effectively mitigate matrix effects and ensure accurate, reliable colorimetric results. Two-dimensional nanomaterials serve as an ideal substrate for constructing highly sensitive and selective colorimetric sensors thanks to their surface-rich, chemically modifiable functional groups, excellent biocompatibility, and the high transparency afforded by their ultrathin sheets [109]. Moreover, the exceptionally large specific surface area of 2DNMs allows them to host a greater density of active species within a limited volume and accelerates interactions between target molecules and sensing elements, thereby markedly enhancing detection sensitivity. At the same time, 2DNM-based colorimetric platforms typically exhibit outstanding structural stability, resistance to oxidation, and reusability, which extend sensor shelf life and ensure stable performance over long-term use [118]. For instance, Li et al. synthesized 2D tungsten trioxide (WO_3_) nanosheets with intrinsic peroxidase-mimicking activity via an environmentally friendly ultrasonic exfoliation technique [119]. These 2D WO_3_ nanosheets exhibited strong catalytic activity in mediating the oxidation of TMB. By immobilizing xanthine oxidase (XOD) onto the nanosheet surface, the researchers developed a xanthine-responsive colorimetric biosensor characterized by high specificity and sensitivity, achieving a detection limit of 1.24 μM and a linear detection range spanning 25–200 μM. In another study, Zhang et al. constructed a 2D/2D heterostructured nanohybrid composed of hexagonal boron nitride (h-BN) and nitrogen-doped molybdenum disulfide (N-MoS_2_), referred to as h-BN/N-MoS_2_, to enhance peroxidase-like catalytic activity for H_2_O_2_ detection [120]. As illustrated in Figure 10a, the heterostructure was fabricated through a combination of liquid-phase ultrasonic exfoliation and solvothermal synthesis. The interface engineering and nitrogen doping modulated the charge carrier distribution and introduced surface defects, which collectively suppressed MoS_2_ restacking, increased the specific surface area, and improved catalytic efficiency. The resulting biosensor demonstrated a wide linear detection range (1–1000 μM) and an impressive detection limit of 0.4 μM.

In a related study, Luan et al. engineered a peptide with the sequence Fmoc-FKKGSHC, which was designed to undergo self-assembly into 2D PNSs under controlled incubation conditions [121]. These PNSs were subsequently functionalized with carboxyl-modified Fe_3_O_4_ magnetic nanoparticles (c-Fe_3_O_4_ MNPs) through electrostatic interactions, resulting in a “switchable” PNSs/c-Fe_3_O_4_ 2D nanomaterial system specifically designed for Hg^2+^ detection. As illustrated in Figure 10b, the detection mechanism relies on the peptide’s thiol (-SH) groups, which, in the absence of Hg^2+^, inhibit the oxidation of TMB, thereby preventing any colorimetric response. Upon the introduction of Hg^2+^ ions, strong Hg^2+^–thiol binding interactions disrupt this inhibition, enabling the catalytic oxidation of TMB and inducing a visible blue color change. This responsive behavior facilitates a highly sensitive and selective colorimetric detection strategy for Hg^2+^ ions.

### 4.2. SPR Biosensors

SPR facilitates the detection and analysis of analytes through the interaction between coordinating ligands and target molecules. Unlike colorimetric biosensors, which rely on chemical reactions and visible color changes, SPR is governed by a physical optical reflection phenomenon. A classic example includes the color change resulting from varying aggregation states of AuNPs in solution. The emerging topic of straintronics in 2DNMs, which investigates the influence of mechanical strain on their physical properties, further underscores the potential of SPR-based optoelectronic applications utilizing 2DNMs [124].

To improve the performance of SPR biosensors, metallic nanostructures are often incorporated to induce localized surface plasmon resonance (LSPR), thereby amplifying the sensitivity of the system [125,126]. Compared with conventional metal-film SPR sensors, the crystal structure, the layer number of the 2DNMs, and the choice of underlying metal all contribute to quantitatively enhanced sensitivity [127,128]. At the same time, by applying mechanical strain to the 2DNMs, the SPR resonance peak can be shifted in wavelength or incident angle; leveraging straintronics, this shift is reversible and tunable, providing strong support for the development of intelligent, biocompatible wearable devices and in-field tunable sensors [129]. In a study conducted by Prajapati et al. [122], antimonene was introduced as a biomolecular recognition element (BRE) for formaldehyde detection. The antimonene surface was biofunctionalized with chitosan to enhance target specificity. As depicted in Figure 10c, a bimetallic Ag/Pt layer was employed, where platinum due to its high melting point, reflectivity, and chemical inertness served both as a protective capping layer for silver and as a sensitivity enhancer. The resulting SPR biosensor exhibited excellent selectivity and stability in formaldehyde detection. During sensing, the chitosan-functionalized BRE selectively captured formaldehyde, which induced a measurable shift in the refractive index of the sensing layer in correlation with the analyte concentration.

In another study, Mujica et al. developed an SPR-based nanosensor for the detection of microRNA-21, a prognostic biomarker for cervical cancer, in urine samples [123]. DNA probes were covalently immobilized onto a bilayer composed of 3-mercaptopropanesulfonate (MPS)-modified gold surfaces and self-assembled layers of poly(diallyldimethylammonium chloride) (PDDA) with GO [123]. As illustrated in Figure 10d, GO played a dual role by anchoring DNA probes and enhancing signal sensitivity through field amplification. This label-free SPR sensing platform demonstrated high sensitivity for miRNA-21, with a linear detection range of 1.0 fM to 10 nM and an exceptionally low detection limit of 0.3 fM. Furthermore, Podila et al. engineered MoS_2_, WS_2_, and h-BN as highly effective dielectric spacer layers on Ag substrates, achieving substantial fluorescence enhancement in RhB via the surface plasmon-coupled emission (SPCE) technique [130]. In the RhB–PVA matrix, the Ag–MoS_2_, Ag–WS_2_, and Ag–BN composite configurations yielded approximately 17-fold, 15-fold, and 9-fold signal amplification, respectively. Notably, the SPCE signal enhancement factor was shown to be tunable based on the refractive index of the spacer layer, underscoring the potential of these nanostructures as versatile platforms for high-sensitivity SPR sensing applications [130].

### 4.3. Electrochemical Biosensors

2DNMs, with their outstanding mechanical strength, flexibility, and optical transparency, have emerged as ideal electrode modifiers for constructing high-performance electrochemical biosensors [131]. Electrochemical sensors, a highly promising analytical platform, typically consist of a biorecognition element, a signal transducer, and a three-electrode system, enabling the highly specific detection of target molecules and finding widespread application in biomarker analysis. By leveraging the high conductivity and exceptionally large specific surface area of 2DNMs, electron transfer kinetics are enhanced and analyte molecules are locally enriched, leading to dramatic improvements in sensitivity and selectivity [132]. Additionally, their layered architecture and tunable surface functionalization strategies confer excellent signal stability under repeated cycling and in complex biological matrices [133]. Furthermore, field-effect transistor sensors based on 2DNMs (2DNM-FETs) offer label-free real-time detection, low-power operation, extreme miniaturization, and arrayed integration, opening new avenues for portable, wearable, and multiplexed clinical diagnostic devices [134].

**Figure 11:**
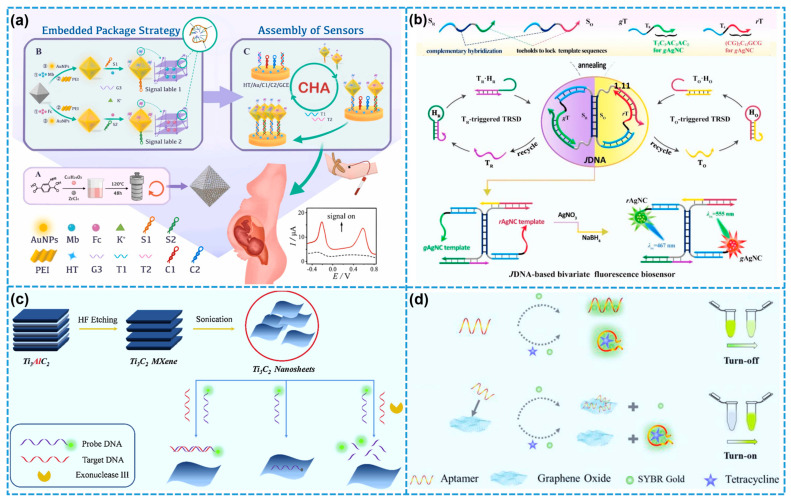
Two-dimensional nanomaterials in electrochemical and fluorescent biosensors: (**a**) MB/Fc-MOFs-G4 as an electrochemical signal for the detection of β-Mediterranean gene mutations CD_41/42_ and CD_17_ in an electrochemical biosensor. Reprinted with permission from ref. [137], Copyright 2025, Elsevier. (**b**) Fluorescent biosensor based on JDNA loaded with dual-emitting Ag nanoclusters (gAgNC and rAgNC). Reprinted with permission from ref. [140], Copyright 2024, Elsevier. (**c**) Single-stranded DNA-modified Ti_3_C_2_ nanosheets for the detection of HPV-18 in a highly sensitive fluorescent biosensing platform schematic. Reprinted with permission from ref. [141], Copyright 2019, Elsevier. (**d**) GO-modulated low background signal and target-induced fluorescence recovery for detection of TC analytes by a highly sensitive fluorescent aptasensor. Reprinted with permission from ref. [142], Copyright 2021, Elsevier.

Among the various biosensing platforms, electrochemical biosensors are often the method of choice for detecting specific cancer biomarkers, owing to their low cost, operational simplicity, high sensitivity, and rapid response [135,136]. As a representative example, Ma and colleagues developed a highly sensitive electrochemical biosensor capable of the simultaneous detection of multiple β-thalassemia gene mutations (specifically CD_41/42_ and CD_17_), which holds great promise for non-invasive prenatal diagnostics (Figure 11a) [137]. The sensor was based on a novel electrochemical labeling approach involving MOFs embedded with G-quadruplex (G4) DNA structures, integrated with a catalytic hairpin assembly (CHA) amplification mechanism. The use of MB/Fc-MOFs-G4 as electrochemical signal tags effectively minimized energy loss and cross-reactivity during electron transfer. The resulting multi-gene electrochemical biosensor exhibited exceptional performance, characterized by ultra-high sensitivity, robust stability, and excellent reproducibility, achieving linear detection over the range of 1.0–500.0 fM and detection limits as low as 0.025 fM for CD_41/42_ and 0.097 fM for CD_17_.

In a study conducted by Chen and collaborators [138], graphdiyne (GDY), a 2D carbon allotrope characterized by a highly π-conjugated structure and excellent dispersibility in aqueous media, was identified as a promising substrate for the construction of electrochemical enzymatic biosensors. Owing to the strong π-π stacking interactions between GDY and bisphenol A (BPA), tyrosinase enzymes were effectively immobilized onto the GDY surface, resulting in the development of a GDY-based electrochemical tyrosinase biosensor. This biosensor exhibited a rapid analytical response, exceptional sensitivity, and superior selectivity for BPA detection, highlighting its potential as a robust and forward-looking analytical tool for environmental monitoring and biosensing applications. In a related study, Lu et al. developed a high-performance electrochemical biosensor for H_2_O_2_ detection by directly electrodepositing gold nanoparticles onto Fe_3_O_4_@ZIF-8-MoS_2_ nanocomposite-modified electrodes [139]. The resulting 2D nanozyme-based sensor demonstrated excellent sensitivity and selectivity under physiological (neutral) conditions. It featured a rapid electrochemical response, a broad linear detection range spanning 5–120 mM, and a low detection limit of 0.9 μM. This nanozyme-functionalized electrode platform offers broad applicability for the sensitive detection of various disease-related biomarkers, thus holding considerable promise for biomedical diagnostics and pharmaceutical evaluation.

### 4.4. Fluorescent Biosensors

The surface chemistry of 2DNMs is highly tunable, facilitating their functional integration with fluorescent materials. Additionally, certain 2DNMs possess intrinsic fluorescence-quenching capabilities, rendering them particularly advantageous for the development of fluorescent biosensors [143]. Like colorimetric and SPR-based platforms, fluorescent biosensors are a subset of optical biosensing technologies.

Fluorescent biosensors transduce biological recognition events into visual signals by monitoring the presence or intensity of variations in the fluorescence, thereby enabling the direct and intuitive detection of target analytes. These sensors are characterized by their high specificity, rapid response times, resistance to magnetic interference, and ease of operation. Furthermore, they exhibit considerable potential for advanced applications in cellular imaging and disease diagnostics or therapeutics [144,145]. For instance, Xu et al. engineered a dual-faced Janus DNA nanoarchitecture (JDNA) functionalized with both green- and red-emitting silver nanoclusters (gAgNCs and rAgNCs) [140]. These bimetallic nanoclusters acted as dual-fluorescent reporters, and the bidirectional programmability of the JDNA scaffold enabled the design of a label-free, dual-signal fluorescent biosensor (Figure 11b). This innovative system offers significant promise for high-sensitivity biosensing applications.

In the study conducted by Peng et al. [141], a highly sensitive fluorescent biosensing platform was established for the detection of human papillomavirus type 18 (HPV-18) by exploiting the differential interactions between Ti_3_C_2_ MXene 2D nanosheets and single-stranded/double-stranded DNA (ssDNA/dsDNA), along with the intrinsic fluorescence-quenching capability of ultrathin Ti_3_C_2_ MXene. As illustrated in Figure 11c, a fluorophore-labeled ssDNA probe was initially adsorbed onto the Ti_3_C_2_ MXene surface via electrostatic interactions, leading to significant fluorescence quenching. Upon the introduction of the target DNA, stronger complementary base pairing between the probe and target DNA induced the desorption of the probe from the MXene surface, thereby restoring fluorescence and enabling effective target detection. Similarly, Xu et al. utilized the interaction between DNA and 2DNMs to develop a GO-based fluorescent aptasensor characterized by a low background signal and target-responsive fluorescence recovery [142]. In the absence of tetracycline-like (TC) analytes, the DNA aptamer was fully adsorbed onto the GO surface, suppressing the fluorescence enhancement of the intercalating dye SYBR Gold (SG) and maintaining a low background. Upon the addition of TC-containing samples, specific binding between the aptamer and TC triggered probe release from the GO surface, facilitating SG intercalation and subsequent fluorescence signal restoration, as shown in Figure 11d.

### 4.5. Photoelectrochemical (PEC) Biosensors

PEC sensing technology has garnered significant interest due to its unique integration of optical excitation with electrochemical detection, enabling low background noise, high sensitivity, and cost-effectiveness. By synergistically combining the advantages of optical and electrochemical techniques, PEC biosensors have been widely applied in diverse fields including energy conversion, early disease diagnostics, food safety, and environmental monitoring [146,147,148].

**Figure 12:**
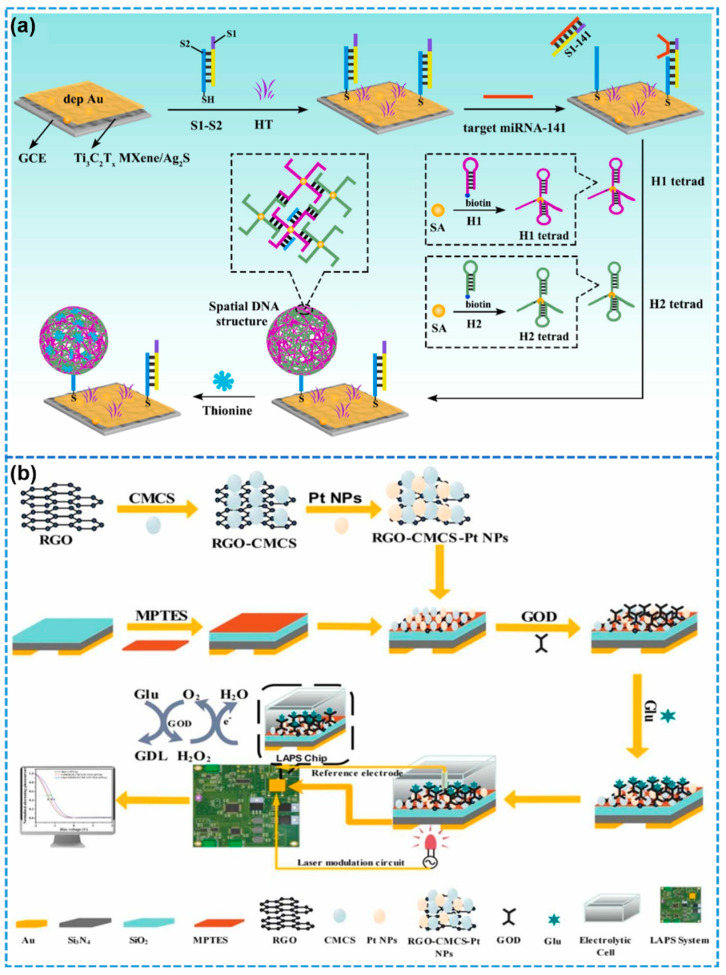
Two-dimensional nanomaterials in photoelectric biosensors: (**a**) Photoelectric biosensor based on double-stranded DNA-modified Ti_3_C_2_ MXene 2D nanosheets. Reprinted with permission from ref. [151], Copyright 2023, Elsevier. (**b**) Glucose photoelectrochemical biosensor modified with RGO-CMCS-Pt NPs. Reprinted with permission from ref. [153], Copyright 2024, Elsevier.4.6. Multimodal biosensors.

In the context of ultra-trace biomarker detection, PEC biosensors exhibit exceptional sensitivity and are particularly well suited for miRNA detection through various signal amplification approaches [149,150]. For example, Huang et al. developed a highly efficient PEC biosensor for the detection of miRNA-141, a critical disease-related biomarker [151]. As depicted in Figure 12a, the biosensing platform employs Ti_3_C_2_ MXene 2D nanosheets as the foundational substrate, onto which Ag_2_S nanocoatings are electrostatically assembled. The modified surface is further functionalized with gold electrodes via Au–S coordination bonds, enabling the stable immobilization of a double-stranded DNA probe (S1–S2). Upon exposure to the target miRNA-141, a strand displacement amplification (SDA) mechanism is activated, leading to the release of the S2 primer strand. This, in turn, initiates a nonlinear hybridization chain reaction (HCR), facilitated by DNA quadruplex structures, resulting in the formation of a spatial DNA nanostructure. The structural amplification significantly enhances photocurrent generation, thereby enabling the highly sensitive detection of miRNA-141.

In a related study, Qu et al. developed a PEC biosensor for the real-time detection of phospholipids in vegetable oils, utilizing a Chox/g-C_3_N_4_TiO_2_ photoactive electrode, where Chox denotes a flavin-dependent cholesterol oxidase enzyme [152]. The integration of Chox significantly improved the specificity and sensitivity of the g-C_3_N_4_/TiO_2_ heterojunction, facilitating rapid, accurate, and user-friendly phospholipid quantification. Similarly, Liang et al. engineered a highly sensitive PEC biosensor for glucose detection by modifying a silicon-based opto-addressable potential sensor with a nanocomposite of reduced GO, carboxymethyl chitosan, and platinum nanoparticles (RGO–CMCS–Pt NPs), as illustrated in Figure 12b [153]. This multifunctional nanocomposite exhibited superior electrical conductivity and strong adsorption capacity, which supported the stable immobilization of GOx. The resulting biosensing interface enabled highly specific and efficient glucose recognition, thereby enhancing the overall performance and sensitivity of the PEC detection platform.

Although single-mode biosensors exhibit substantial sensing performance, their effectiveness in detecting ultra-trace levels of biomarkers remains limited. To overcome these challenges, dual-mode biosensors based on hybrid nanomaterials integrating modalities such as optical–optical, optical–electrochemical, or electrochemical–electrochemical have gained significant attention. These multifunctional platforms enhance detection accuracy, sensitivity, and reliability, positioning them as pivotal technologies for the precise identification of a wide range of biomarkers [154].

**Figure 13:**
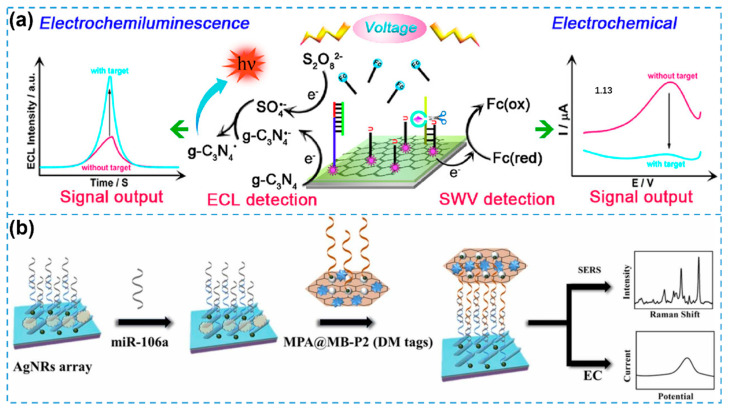
Two-dimensional nanomaterials in multimodal biosensors: (**a**) Schematic diagram of an ECL and EC dual-mode biosensor platform based on a self-powered DNAzyme walker with g-C_3_N_4_. Reprinted with permission from ref. [156], Copyright 2023, American Chemical Society. (**b**) Schematic diagram of mF-MoS_2_-based SERS/electrochemical dual-mode biosensor platform for the detection of gastric-cancer-associated miR-106a. Reprinted with permission from ref. [157], Copyright 2022, Elsevier.

By capitalizing on the high specific surface area, tunable surface chemistry, and exceptional biocompatibility of 2DNMs, researchers have engineered multifunctional hybrid nanostructures that integrate the advantageous physicochemical properties of various nanomaterials. This approach facilitates the development of dual-functional 2D nanohybrids for multimodal biosensing applications [155]. For instance, Du et al. reported a self-powered DNAzyme walker-based biosensor platform combining electrochemiluminescence and electrochemical modalities for the accurate and sensitive detection of miRNA-21 [156]. As shown in Figure 13a, this system harnessed a Mg^2+^-dependent DNAzyme cleavage reaction. DNAzyme walkers and ferrocene (Fc)-labeled substrate strands were immobilized onto gold nanoparticles deposited on a g-C_3_N_4_-coated electrode. The dual-mode g-C_3_N_4_-based biosensor exhibited high performance in both electrochemiluminescence and electrochemical detection modes. Upon the introduction of target miRNA-21 and Mg^2+^, the DNAzyme walker was activated, cleaving the Fc-labeled substrates and releasing multiple Fc molecules. Because Fc quenches the photoelectrochemical activity of g-C_3_N_4_, its release led to a simultaneous restoration of the ECL signal and suppression of the EC signal. This complementary signal modulation effectively reduced cross-reactivity and enhanced detection accuracy, making the platform highly suitable for early disease diagnosis and biomarker monitoring. In another study, Song et al. developed an in situ dual-mode biosensor for the detection of gastric-cancer-associated microRNA-106a (miR-106a), employing multifunctional molybdenum disulfide (mF-MoS_2_) probes and a surface-enhanced Raman scattering (SERS)-active silver nanorod (AgNRs) array electrode (Figure 13b) [157]. This biosensing system demonstrated outstanding selectivity and sensitivity for cancer biomarker detection, highlighting its potential for clinical diagnostic applications [157].

In the realm of emerging analytical technologies, SERS has established itself as a unique and powerful technique capable of providing distinct spectral fingerprints at the single-molecule level [158,159]. Compared to conventional analytical methods, SERS-based sensors offer significant advantages, including cost-effectiveness, exceptional sensitivity, and superior stability, enabling the detection of ultra-trace levels of biomarkers as well as simultaneous multi-analyte analysis [160]. These attributes make SERS-based platforms highly promising for applications in environmental monitoring and medical diagnostics [161]. Moreover, Raman spectroscopy inherently supports label-free molecular detection, and the incorporation of 2DNMs—owing to their intrinsic Raman activity and unique physicochemical properties—can significantly enhance the sensitivity and specificity of 2DNM-based SERS biosensors [162,163,164]. For instance, Kim et al. developed a dual-mode electrochemical/SERS biosensing platform for the detection of Middle East respiratory syndrome coronavirus nanovesicles (MERS-NVs), utilizing a multifunctional DNA three-way junction aptamer (MF aptamer) in combination with GO-MoS_2_ nanocomposite [165]. The platform integrated the electrochemical impedance spectroscopy (EIS) and SERS techniques. The GO-MoS_2_ composite not only facilitated efficient charge transfer to enhance the EIS response but also amplified the Raman signal. Covalent interactions were employed to immobilize the MF aptamer on the surface of the GO-MoS_2_. The resulting gold-substrate-based sensor demonstrated remarkable detection performance, achieving a SERS detection limit of 0.176 pg/mL and an EIS detection limit of 0.405 pg/mL in PBS. Furthermore, the sensor maintained excellent performance in 10% human saliva, with detection limits of 0.525 pg/mL (SERS) and 0.645 pg/mL (EIS), highlighting its strong potential for rapid, label-free virus diagnostics in complex biological matrices.

## 5. Conclusions and Outlook

Two-dimensional nanomaterials have emerged as a versatile and powerful class of materials for biosensor development due to their unique layered structures, excellent physicochemical properties, and high surface area for functionalization. In this review, we systematically summarized recent progress in the biomodification and biomimetic synthesis of 2DNM-based nanohybrids, with particular emphasis on their integration into high-performance biosensing platforms. We discussed various biomacromolecule-based functionalization strategies and biomimetic synthesis methodologies that enable precise structural control, enhanced biocompatibility, improved physicochemical stability, and targeted molecular recognition. The convergence of biofunctionalized 2DNMs with diverse sensing modalities ranging from colorimetric, electrochemical, and fluorescence sensors to SPR and photoelectrochemical platforms has significantly expanded the sensitivity, specificity, and applicability of biosensors. These advances are critical for rapid bioanalysis, early disease diagnostics, and real-time environmental monitoring.

Despite significant advancements, several critical challenges and opportunities remain in the development of 2DNM-based biosensors. One key direction involves the pursuit of sustainable and scalable synthesis methodologies that minimize environmental impact while enabling industrial and biomedical deployment. Equally important is the engineering of stimuli-responsive smart materials based on 2DNMs, which can dynamically respond to environmental factors such as pH, temperature, or biomolecular signals. Additionally, integrating multiple sensing modalities into compact, user-friendly platforms will be vital to enhance analytical performance, enable real-time monitoring, and meet the demands of point-of-care diagnostics.

Another emerging frontier lies in the advanced biofunctionalization of 2DNMs using tools from gene editing and synthetic biology. For example, CRISPR/Cas complexes can be immobilized on 2D surfaces such as MoS_2_ or graphene oxide to enable site-specific nucleic acid targeting with ultra-high selectivity. Meanwhile, cell-free protein synthesis offers a rapid and programmable method to generate functional peptides or enzymes that impart novel capabilities, such as self-healing or degradability. However, ensuring the long-term stability, biocompatibility, and in vivo performance of these biofunctionalized systems remains a crucial challenge. Addressing this requires a combination of standardized accelerated aging models, in vivo imaging, and omics-based toxicity assessments to validate biosensor safety and efficacy under physiological conditions.

Furthermore, the rational selection and precise control of the biomolecules used for 2DNM surface modification are critical for maximizing biosensor performance. The choice between peptides, proteins, and nucleic acids should be guided by the application context, compatibility with the 2DNM surface, and functional stability in target environments. While peptides offer modular design and ease of self-assembly, proteins provide high specificity, and DNA/RNA enable programmable recognition. Additionally, surface density and molecular orientation—adjusted through covalent coupling strategies or self-assembled monolayers—can significantly affect biocompatibility, catalytic efficiency, and analyte accessibility. A deeper understanding of these molecular interactions will inform the design of next-generation biosensors. Ultimately, overcoming translational barriers—such as the absence of standardized protocols for biosafety, reproducibility, and functional validation, alongside complex regulatory pathways and uncertain biostability in real-world settings—will require modular design strategies, simulation-driven reliability testing, and robust biointerface engineering. These approaches will be key to bridging the gap from laboratory innovation to practical, real-world applications that benefit human health and promote environmental sustainability, ultimately realizing the full potential of 2DNM-based biosensing technologies.

## Figures and Tables

**Table 1:** The modification strategies of 2DNMs by various biomaterials.

Biomaterials	2DNMs	Interaction	Ref
Peptides	GO	Non-covalent interactions	[45]
Peptides	g-C_3_N_4_	Non-covalent interactions	[46]
Peptides	GO/MoS_2_	Non-covalent interactions	[47]
Peptides	GO	Covalent interactions	[79]
Proteins	MOF [MIL-53 (Al)]	Non-covalent interactions	[48]
Proteins	Ti_3_C_2_ MXene	Covalent interactions	[49]
Proteins	BP	Non-covalent interactions	[53]
Proteins	Composite nanofibrous sheets	Non-covalent interactions	[78]
RNA	GO	Non-covalent interactions	[55]
RNA	MOF	Non-covalent interactions	[80]
DNA	COF	Covalent interactions	[56]
DNA	GO	Non-covalent interactions	[57]
Bio-enzymes	TiS_2_	Non-covalent interactions	[58]
Bio-enzymes	Ti_3_C_2_ MXene	Non-covalent interactions	[59]
Bio-enzymes	GO	Non-covalent interactions	[60]
Bio-enzymes	MOF	Non-covalent interactions	[61]
Biopolymers	BP	Non-covalent interactions	[62]
Biopolymers	GO/MoS_2_	Non-covalent interactions	[63]
Biopolymers	MXene	Non-covalent interactions	[68]
Bioactive polysaccharides	GO	Covalent interactions	[69]
Bioactive polysaccharides	BP	Non-covalent interactions	[77]

**Table 2:** Summary of representative biomimetic synthesis strategies of 2DNMs.

Strategy	Template/Mediator	Material/Function	Ref
Bio-templating	Collagen	Protein–manganese phosphate nanoflowers	[86]
Bio-templating	Cabbage leaf cells	2D manganese oxide nanosheets	[87]
Bio-templating	Hydrophobic peptides	2D ZnO nanosheets	[88]
Biomolecule-directed self-assembly	Fmoc-FKKGSH sequence peptides	2D PNS/PEG-Ag_2_S QDs	[92]
Biomolecule-directed self-assembly	Fmoc-FKKGSHC sequence peptides	AuNPs/PNS	[93]
Biomolecule-directed self-assembly	KIIIIKYWYAF sequence peptides	BMNS-PNF	[94]
Biomolecule-directed self-assembly	DNA	DNA origami structures	[95]
Biomineralization strategies	PNFs and CNFs	GO/PNF/CNF-ZrO_2_ composite	[100]
Biomineralization strategies	CaP	GA-CaP-GO/DOX/siRNA intelligent drug delivery system	[101]
Biomineralization strategies	Albumin	PZM composite scaffold	[102]
Biomimetic functional integration	Hemin molecules	2DNM HBW	[104]
Biomimetic functional integration	Cardanol	Bio-based nanocomposites	[105]
Biomimetic functional integration	GO/MXene	Biomimetic wood-like coaxial fibers	[106]
Biomimetic functional integration	Hierarchical micro-/nanostructures in lotus leaves	MoS_2−x_Se_x_ alloy nanocomposites	[107]

## Data Availability

Not applicable.
